# Large-Scale Synthesis of the Stable Co-Free Layered Oxide Cathode by the Synergetic Contribution of Multielement Chemical Substitution for Practical Sodium-Ion Battery

**DOI:** 10.34133/2020/1469301

**Published:** 2020-10-19

**Authors:** Yao Xiao, Tao Wang, Yan-Fang Zhu, Hai-Yan Hu, Shuang-Jie Tan, Shi Li, Peng-Fei Wang, Wei Zhang, Yu-Bin Niu, En-Hui Wang, Yu-Jie Guo, Xinan Yang, Lin Liu, Yu-Mei Liu, Hongliang Li, Xiao-Dong Guo, Ya-Xia Yin, Yu-Guo Guo

**Affiliations:** ^1^CAS Key Laboratory of Molecular Nanostructure and Nanotechnology, CAS Research/Education Center for Excellence in Molecular Sciences, Beijing National Laboratory for Molecular Sciences (BNLMS), Institute of Chemistry, Chinese Academy of Sciences (CAS), Beijing 100190, China; ^2^School of Chemical Engineering, Sichuan University, Chengdu 610065, China; ^3^Institute of Materials for Energy and Environment, College of Materials Science and Engineering, Qingdao University, Qingdao 266071, China; ^4^University of Chinese Academy of Sciences, Beijing 100049, China; ^5^Beijing National Laboratory for Condensed Matter Physics, Institute of Physics, Chinese Academy of Sciences (CAS), Beijing 100190, China

## Abstract

The O3-type layered oxide cathodes for sodium-ion batteries (SIBs) are considered as one of the most promising systems to fully meet the requirement for future practical application. However, fatal issues in several respects such as poor air stability, irreversible complex multiphase evolution, inferior cycling lifespan, and poor industrial feasibility are restricting their commercialization development. Here, a stable Co-free O3-type NaNi_0.4_Cu_0.05_Mg_0.05_Mn_0.4_Ti_0.1_O_2_ cathode material with large-scale production could solve these problems for practical SIBs. Owing to the synergetic contribution of the multielement chemical substitution strategy, this novel cathode not only shows excellent air stability and thermal stability as well as a simple phase-transition process but also delivers outstanding battery performance in half-cell and full-cell systems. Meanwhile, various advanced characterization techniques are utilized to accurately decipher the crystalline formation process, atomic arrangement, structural evolution, and inherent effect mechanisms. Surprisingly, apart from restraining the unfavorable multiphase transformation and enhancing air stability, the accurate multielement chemical substitution engineering also shows a pinning effect to alleviate the lattice strains for the high structural reversibility and enlarges the interlayer spacing reasonably to enhance Na^+^ diffusion, resulting in excellent comprehensive performance. Overall, this study explores the fundamental scientific understandings of multielement chemical substitution strategy and opens up a new field for increasing the practicality to commercialization.

## 1. Introduction

Owing to the environmental pollution and political unrest with regard to fossil fuel production, electrochemical energy storage and conversion (EESC) technology with low cost, high Tefficiency, long lifespan, and adequate safety is important [1–8]. Sodium-ion batteries (SIBs) are considered as a smart choice to efficiently improve the grid reliability and utilization due to the abundant reserves and low cost as well as similar insertion mechanism to the commercial lithium-ion battery [9–15].

As cathode materials are the key determinant for SIBs, a large variety of cathode materials have been mostly studied so far [16–20]. Among the future leading practical application candidates, O3-type oxide cathodes NaTMO_2_ (where TM is a first-row transition metal) have aroused considerable interest due to their high initial Coulombic efficiency, excellent ionic conductivity, and good environmental benignity [21–23]. However, some major problems are obstructing progress toward their commercial application: (1) As all know, cobalt element could boost transport kinetics, while the concept of no cobalt-containing components in O3-type cathodes becomes more and more important in EESC because of the toxicity and insufficient supply as well as rising costs of cobalt [24, 25]. (2) Although O3-type NaNi_0.5_Mn_0.5_O_2_ cathodes have the advantage of being cobalt-free, they normally show poor air stability and exhibit notable performance losses, which originate from the formation of electrochemically inactive NaOH or Na_2_CO_3_ [26, 27]. Meanwhile, these alkaline species would induce the defluorination of polyvinylidene fluoride binder and corrosion of the current collector, which results in particle agglomeration and slurry gelation when preparing the working electrode [28, 29]. (3) The frequent phase transformations of O3-type NaNi_0.5_Mn_0.5_O_2_ cathodes accompanied by multiple voltage plateaus in the electrochemical curves, such as O3_hex._-O3′_mon._-P3_hex._-P3′_mon._-P3′′_hex._, are inclined to result in rapid capacity decline and the sluggish kinetics as well as poor rate performance [30]. Therefore, improving the comprehensive performance of the Co-free NaNi_0.5_Mn_0.5_O_2_ cathode materials through simultaneously enhancing the air stability, reducing or suppressing the irreversible phase transition, and alleviating the sluggish kinetics is of great importance to realize sodium-ion battery commercialization for market applications. According to the reported works, chemical substitution strategy could modulate the physical as well as chemical properties and indeed enhance comprehensive performance [31–35]. However, the accurate multielement chemical substitution engineering is still a big challenge in large-scale production to obtain a high rate and superlong cycle performance to meet the requirements of practical applications. Meanwhile, a thorough and in-depth systematic investigation of the structure–function–property relationship is needed, with an emphasis placed on the formation process, atomic arrangement, structural evolution, and electrochemical intercalation/deintercalation behavior, which could offer new insights into the rational design of high-performance battery cathode materials.

Hence, a stable Co-free O3-type NaNi_0.4_Cu_0.05_Mg_0.05_Mn_0.4_Ti_0.1_O_2_ cathode material for practical SIBs was synthesized by a facile high-temperature solid-state method with large-scale production. By virtue of partially substituting copper and magnesium into the nickel sites and titanium into the manganese sites, this novel layered oxide cathode not only shows excellent physical and chemical properties but also delivers outstanding cycle performance (76.4% after 1000 cycles at 5C) and excellent capacity retention of 73.6% (10C compared to 0.2C) in half-cell system and superior battery performance in full-cell system. Meanwhile, the inherent synergetic contributions of rationally selected multielement chemical substitution engineering strategy are clearly articulated and confirmed by various *in situ* X-ray diffraction (XRD) and multiscale scanning transmission electron microscopy as well as other advanced characterization techniques. All these results indicate that this novel compound promises to be a very competitive cathode material for future large-scale practical application.

## 2. Results

**Figure 1:**
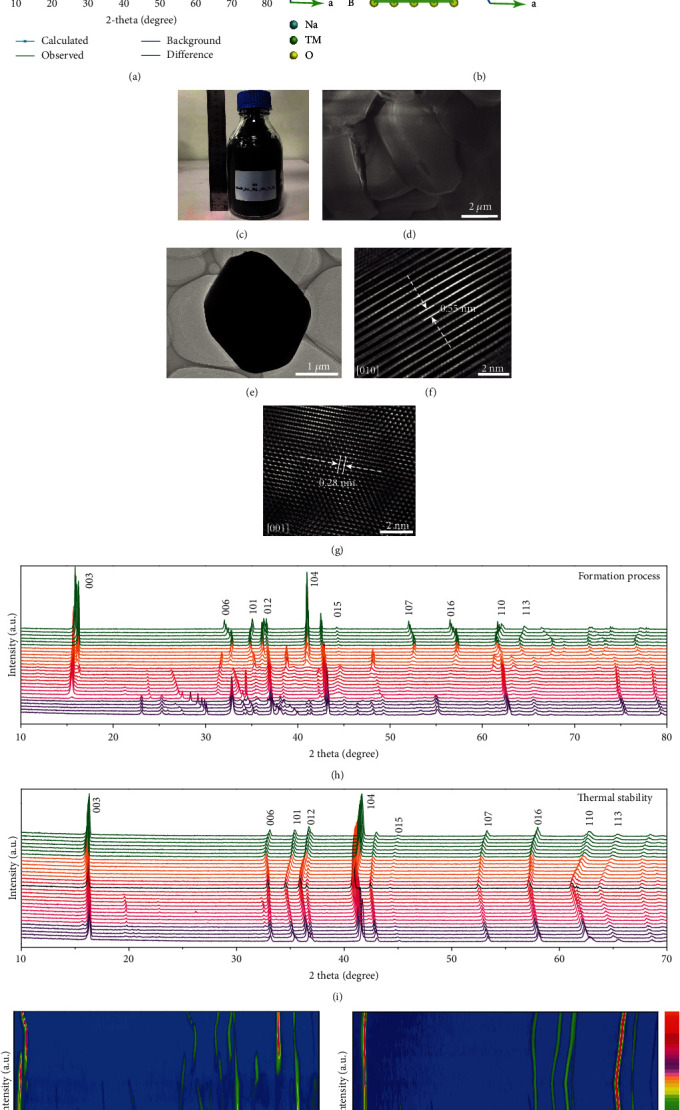
Crystal structure of O3-NaNCMMT cathode material. (a) Powder XRD pattern and Rietveld refinement plot. (b) Crystal structures viewed along the (010) and (001) crystallographic directions. (c) Optical photograph. (d) SEM image. (e) TEM image. (f, g) HR-TEM images. (h–k) *In situ* HRXRD patterns of the formation process and thermal stability as well as corresponding intensity contour maps (bird's eye view) concerning the evolution of the characteristic diffraction peaks.

The crystal structure of O3-type NaNi_0.4_Cu_0.05_Mg_0.05_Mn_0.4_Ti_0.1_O_2_ cathode material (hereafter denoted as O3-NaNCMMT) is ascribed to the *α*-NaFeO_2_ layered structure with rhombohedral R$\bar{3}$m space group, containing traces of NiO crystalline impurity (Figure 1(a)). Meanwhile, the detailed crystallographic parameters and atomic site occupations of the Rietveld refinement (*R*_wp_ 6.84% and *R*_p_ 4.77%) are displayed in Table S1–S2 (Supporting Information), and the results show that copper, magnesium, and titanium elements localize at the octahedral 3b Wyckoff sites. Owing to the similar ionic radii and same valence (Ni^2+^ = 0.69 Å, Cu^2+^ = 0.73 Å, Mg^2+^ = 0.72 Å, Mn^4+^ = 0.53 Å, and Ti^4+^ = 0.605 Å), the uniform incorporation of multielement chemical substitution into the TM layers could effectively modulate the crystal structure and alleviate the lattice strains driven by the large Ni ionic radius change during Na^+^ intercalation/deintercalation, resulting in better structural stability [30, 36]. Moreover, compared with the crystallographic parameter (*c* = 16.0301 Å) of Cu and Ti cosubstituted NaNi_0.45_Cu_0.05_Mn_0.4_Ti_0.1_O_2_ sample, the substitution of Mg^2+^ in O3-NaNCMMT cathode material not only shows a pinning effect but also enlarges the interlayer spacing reasonably (*c* = 16.0812 Å), which could improve the Na^+^ diffusion coefficient and contribute to the high rate capability, as discussed later [37]. In typical O3-type layered structure, sodium ions are accommodated at the octahedral sites between TMO_2_ layers, where TMO_2_ layers duplicate in the manner of *αβγαβγ* with the ABCABC-stacking mode of oxygen columns (Figure 1(b) and Figure S1, Supporting Information) [38]. As illustrated in Figures 1(c)–1(e) and Figure S2 (Supporting Information), the well-defined layered cathode material revealed in the scanning electron microscopy (SEM) and transmission electron microscopy (TEM) images could be obtained through large-scale production (>2 kg per batch). The hexagonal symmetry structure of the O3-type layered phase was also confirmed by the lattice spacing reasonably (0.55 nm and 0.28 nm) of high-resolution transmission electron microscopy (HR-TEM) and the fast Fourier transform (FFT) (Figures 1(f) and 1(g), and Figure S3-S4, Supporting Information). Besides, *in situ* high-energy XRD (HEXRD) was executed to accurately decipher the formation process and the structure change as well as thermal stability of O3-NaNCMMT cathode material in air atmosphere from room temperature to 1000°C (Figures 1(h) and 1(i) and Figure S5-S6, Supporting Information) [39]. It is worth noting that the different colour regions stand for the different calcination temperature, which shows the detailed formation evolution process. Moreover, a good 2D contour map of the formation process and thermal stability of O3-NaNCMMT cathode material is afforded by further analysis on the data (Figures 1(j) and 1(k), and Figure S7, Supporting Information). As the temperature increases, all the XRD reflections (for example, (003), (006), (101), (012), and (104)) reveal continuous peak shift toward a lower angle because of the lattice expansion, and the peaks completely recover to the original positions after cooling to room temperature, suggesting excellent thermal stability. Besides, the results of chemical compositions analyzed by inductively coupled plasma mass spectrometry (ICP-MS) are listed in Table S3 (Supporting Information).

**Figure 2:**
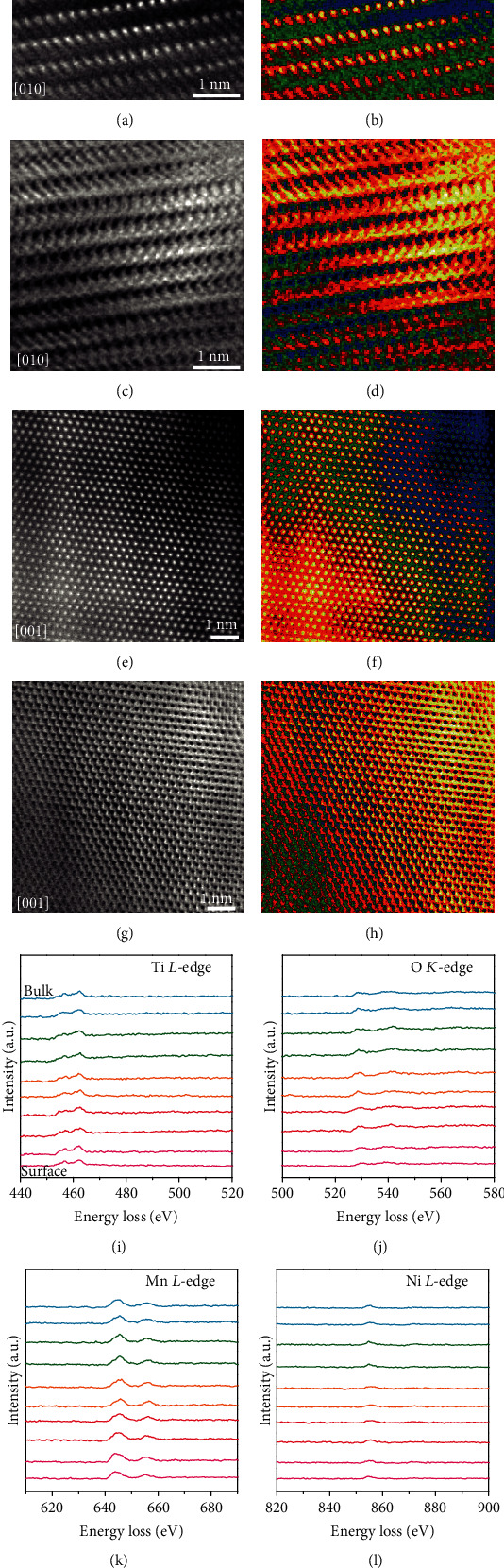
Atomic structure of O3-NaNCMMT cathode material. (a–d) HAADF and ABF-STEM images and the corresponding colored patterns viewed along the (010) crystallographic direction. (e–h) HAADF and ABF-STEM images and the corresponding colored patterns viewed along the (001) crystallographic direction. (i–l) EELS spectra of Ti L-edges, O K-edge, Mn L-edges, and Ni L-edges scanned from the surface to the bulk with an increment of 2 nm per spectrum.

The contrast of the high-angle annular dark-field (HAADF) image of scanning transmission electron microscopy (STEM) exhibits an approximately Z^1.7^ dependence with respect to the atomic number Z. The dark dots in the annular bright field- (ABF-) STEM images and the white dots in the HAADF-STEM images reveal the transition metal atom positions [40–46]. In addition, the faint dark spots stand for the Na and O light elements in the ABF-STEM. As shown in Figures 2(a)–2(d) and Figure S8 (Supporting information), the atomic arrangements of O3- type layered phase are identical to the atomic configuration viewed along the (010) crystallographic direction. Meanwhile, as displayed in Figures 2(e)–2(h), the results of HAADF and ABF-STEM images with the corresponding colored patterns as well as FFT patterns match well with the atomic packing model viewed along the (001) crystallographic direction (Figure S9, Supporting Information). To accurately evaluate the chemical composition, the O3-NaNCMMT sample was scanned from the surface to the center with an increment of 2 nm by electron energy loss spectroscopy (EELS), and the corresponding spectra were simultaneously collected (Figures 2(i)–2(l) and Figure S10, Supporting Information). Energy loss peaks of Ti L-edge, O K-edge, Mn L-edge, and Ni L-edge are located in the range of 440-480 eV, 520-560 eV, 630-670 eV, and 840-880 eV, respectively [47, 48]. It is worth noting that all the detected elements are homogeneously distributed in the bulk and surface without structure distortion. Based on these finds mentioned above, it can be deduced that a novel Co-free O3-type layered NaNi_0.4_Cu_0.05_Mg_0.05_Mn_0.4_Ti_0.1_O_2_ cathode material has been successfully fabricated by high-temperature solid-state reaction with large-scale production.

**Figure 3:**
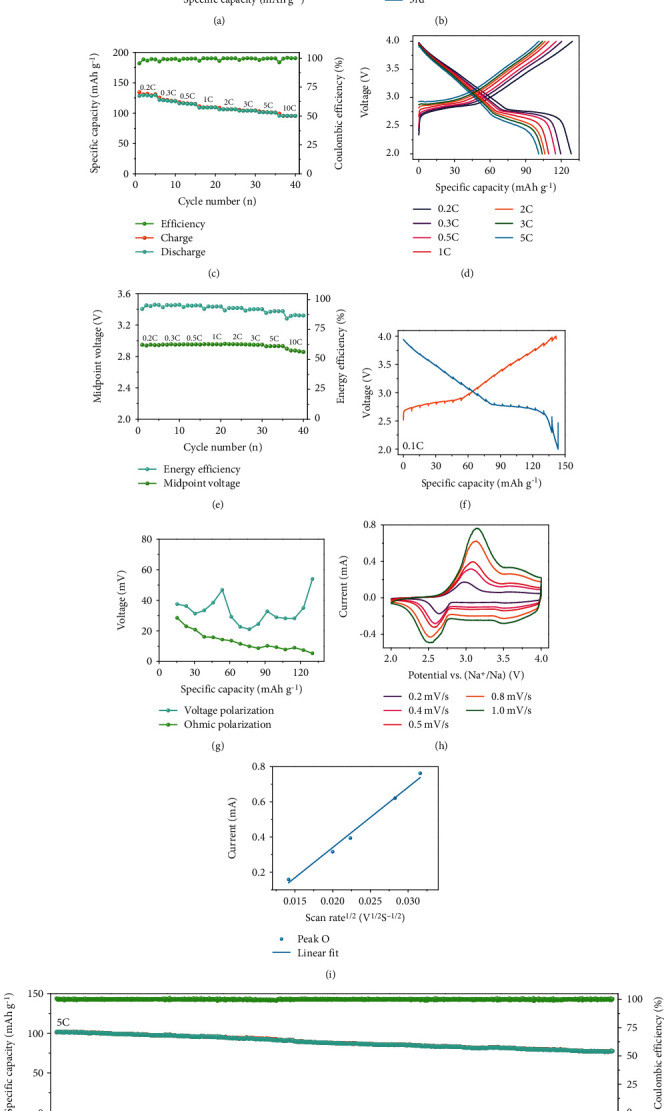
Electrochemical performance of O3-NaNCMMT electrode. (a) Galvanostatic charge/discharge curves versus specific capacity at 0.2C in the voltage range of 2.0-4.0 V. (b) Cyclic voltammograms at 0.1 mV s^−1^ in different cycles. (c, d) Rate performance at various rates and corresponding galvanostatic charge/discharge curves versus specific capacity. (e) Midpoint voltage and energy efficiency. (f, g) GITT curves and corresponding voltage polarization with ohmic polarization. (h, i) Cyclic voltammograms at different scan rates and corresponding linear fitting of peak current versus square root of the scan rate at different oxidation peaks. (j) Cycling performance during 1000 cycles at 5C after performance tests at various rates.

To evaluate the electrochemical properties of O3-NaNCMMT electrodes, galvanostatic charging-discharging experiments were performed in Na half cells. The specific capacity and energy density of O3-NaNCMMT electrode are 129.7 mAh g^−1^ and 397.9 Wh kg^−1^, respectively, at 0.2C within 2.0–4.0 V, and the first three cyclic voltammetry (CV) curves overlap well (Figures 3(a) and 3(b), and Figure S11a, Supporting Information). Meanwhile, the O3-NaNCMMT electrode displays outstanding electrochemical performance, delivering a capacity retention of 73.6% (10C compared to 0.2C) and exhibiting stable Coulombic efficiency and voltage as well as excellent energy efficiency at different rates (Figures 3(c)–3(e) and Figure S11b, Supporting Information). It is worth noting that the capacity retention could recover to 101.5% (110.8 mAh g^−1^ compared to 109.2 mAh g^−1^) after the rate comes back to 1C (Figure S11c, Supporting Information). Furthermore, detailed electrochemical parameters at each current density are displayed in Table S4 (Supporting Information). The continuous change of equilibrium voltage above 2.7 V reveals a solid-solution reaction process, and small ohmic polarization with voltage polarization is observed through the galvanostatic intermittent titration technique (GITT) (Figures 3(f) and 3(g)). The high Na^+^ apparent diffusion coefficient (1.578 × 10^–11^ cm^2^ s^–1^) of O3-NaNCMMT electrode is calculated from the CV (Figures 3(h) and 3(i), and Table S5, Supporting Information). Besides, the CV of the O3-NaNCMMT electrode resembles the property of capacitors, and the detailed calculation process of electrochemical behavior is displayed in Figure S11d and Figure S12 (Supporting Information) [49, 50]. An electrochemical impedance spectroscopy (EIS) technique was also executed to obtain the charge transfer resistance and Warburg impedance (Figure S13, Supporting Information). After performance tests at various rates, the O3-NaNCMMT electrode shows an outstanding capacity retention (76.4% after 1000 cycles at 5C) with an excellent energy efficiency and a stable midpoint voltage (Figure 3(j), Figure S14, and Figure S15a, b, Supporting Information). It is worth mentioning that the O3-NaNCMMT electrode still shows a superior capacity retention of 65.6% after 1500 cycles at 5C (Figure S15c and S16, Supporting Information). Overall, compared with the unsubstituted NaNi_0.5_Mn_0.5_O_2_ and Ti substituted NaNi_0.5_Mn_0.2_Ti_0.3_O_2_ as well as Cu and Ti cosubstituted NaNi_0.45_Cu_0.05_Mn_0.4_Ti_0.1_O_2_ samples, the O3-NaNCMMT cathode material delivers better rate performance and cycling stability (Table S6, Supporting Information).

**Figure 4:**
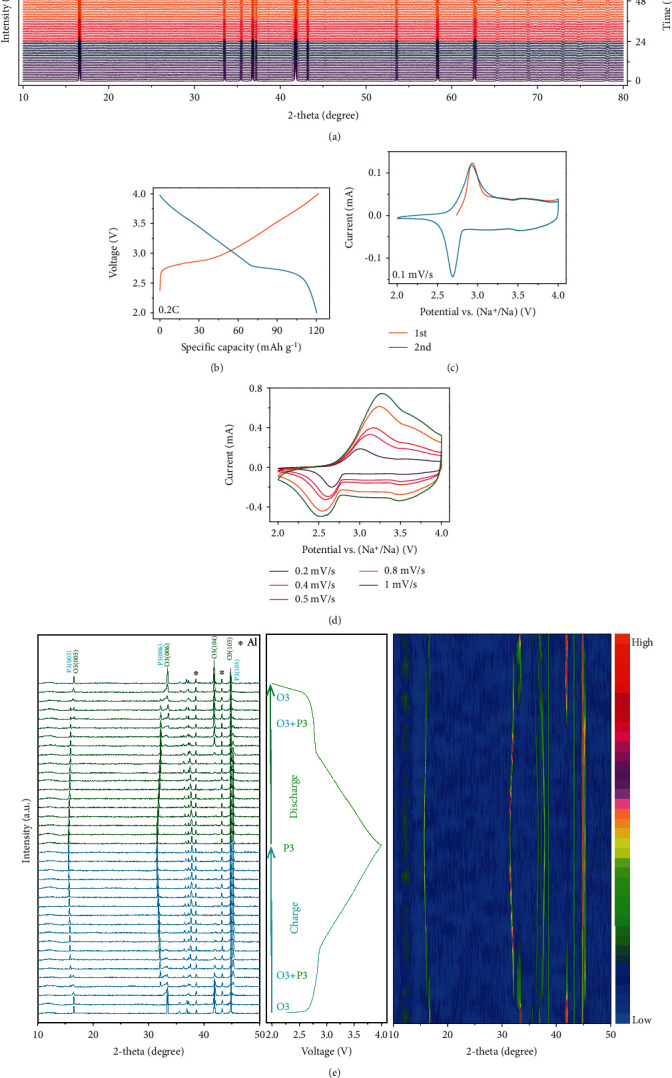
Air-exposure stability and crystal structure evolution under Na^+^ (de)intercalation. (a) In situ XRD patterns of air-exposure stability test for three days (the different colour regions stand for the different air-exposure stages). (b–d) Galvanostatic charge/discharge curves versus specific capacity at 0.2C and cyclic voltammograms at 0.1 mV s^−1^ in different cycles as well as cyclic voltammograms at different scan rates after exposing O3-NaNCMMT cathode material to air for three days. (e) In situ XRD patterns during the charge/discharge process at 0.1C in the voltage range of 2.0-4.0 V and corresponding intensity contour maps (bird's eye view) concerning the evolution of the main characteristic diffraction peaks.

To test the air stability of O3-NaNCMMT cathode material, *in situ* XRD measurement of the aging experiment was carried out (Figure 4(a)). According to the previous work, the structure change of conventional O3-type cathode material is already observed after 2 h of air exposure while the Na-deficient phase gradually increases as the prolongation of air-exposure time [37, 51]. In contrast, no extra peaks beyond the O3-type phase can be observed, and the intensity of the different peaks maintains consistency after exposing O3-NaNCMMT cathode materials to air for 3 days. Besides, as shown in Figures 4(b) and 4(c), the galvanostatic charge/discharge curve and CV test of aging O3-NaNCMMT show similar results without affecting the specific capacity and electrochemical behavior. Furthermore, the high Na^+^ apparent diffusion coefficients and the mixed-contribution controlled sodium storage mechanism of aging O3-NaNCMMT are also demonstrated via quantitative electrochemical kinetics calculation (Figure 4(d) and Figure S17-S18 and Table S7, Supporting Information). Moreover, to further unravel the detailed structure evolution mechanism of the O3-NaNCMMT electrode, *in situ* XRD experiment during the charge/discharge process was carried out (Figure 4(e) and Figure S19, Supporting Information) [52]. As Na^+^ is being extracted, (001) peaks shift to a lower angle and then split into two, revealing that the new P3 phase starts to form and the intensity of (104)_O3_ peak is clearly reduced *via* an O3–P3 two-phase reaction [53, 54]. When subsequent sodium continues to be removed, the peaks of the P3 phase shift toward a lower angle without the appearance of any new peaks until charged to 4 V, indicating a solid-solution reaction. During the discharging process, the structure experiences an exact opposite evolution, which accounts for the superior battery performance. Furthermore, combining in situ XRD results and the corresponding Rietveld refinement, the results show small unit cell volume change and low-strain characteristics before and after Na extraction, which is responsible for the excellent cycling stability (Figure S20, Supporting Information). Overall, air stability, multiphase evolution, and cycling lifespan are tackled concurrently by virtue of the accurate multielement chemical substitution strategy. Namely, the rationally selected multielement chemical substitution of Ti for Mn and Cu for Ni could not only increase the ionicity of the crystal lattice and the redox potential as well as restrain the unfavorable multiphase transformation but also improve the air stability because of the suppression of spontaneous Na extraction and enhanced antioxidizability. Furthermore, the inactive magnesium element also shows a pinning effect in improving the high structural reversibility and enlarges the interlayer spacing reasonably to enhance rate capability [55–57].

**Figure 5:**
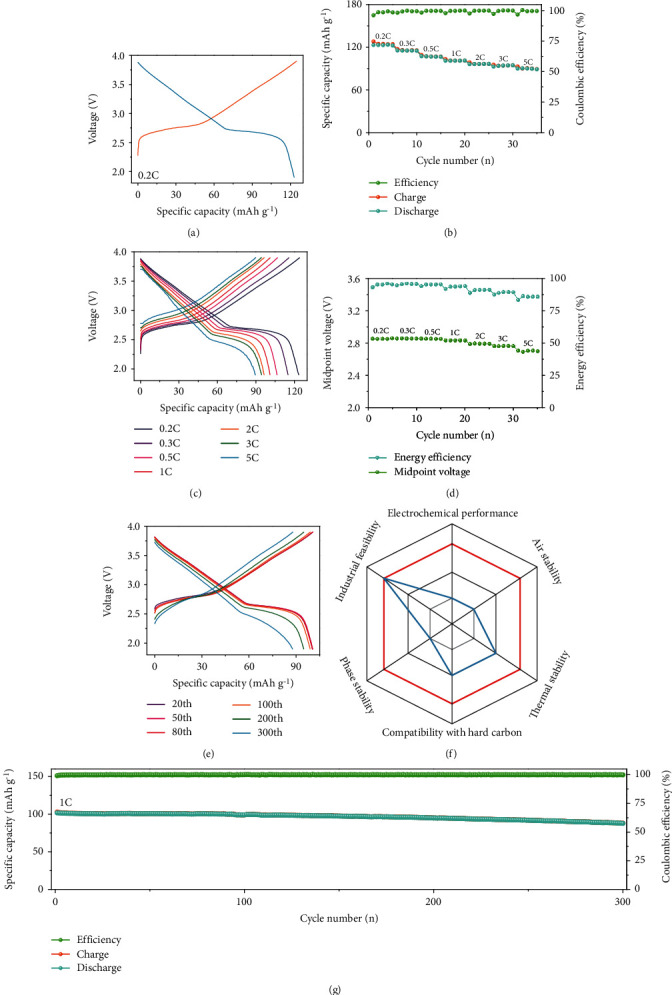
Electrochemical performance of full-cell system. (a) Galvanostatic charge/discharge curves versus specific capacity at 0.2C in the voltage range of 1.9-3.9 V. (b, c) Rate performance at various rates and corresponding galvanostatic charge/discharge curves versus specific capacity. (d) Energy efficiency and midpoint voltage at various rates in the voltage range of 1.9-3.9 V. (e) Galvanostatic charge/discharge curves versus specific capacity in different 20th, 50th, 80th, 100th, 200th, and 300th cycles at 1C. (f) The comparison of the comprehensive performance of the NaNi_0.4_Cu_0.05_Mg_0.05_Mn_0.4_Ti_0.1_O_2_ (red line) and NaNi_0.5_Mn_0.5_O_2_ (blue line) cathode materials. (g) Cycling performance during 300 cycles at 1C.

The practicability of O3-NaNCMMT cathode material was confirmed by the test of full-cell system. The presodiation of the hard carbon anode was conducted by an electrochemical process (Figure S21, Supporting Information) [58–60]. The full-cell displays a high capacity of 123.4 mAh g^−1^ at 0.2C corresponding to energy density of 367.4 Wh kg^−1^ based on the mass of cathode (Figure 5(a) and Figure S22, Supporting Information) and shows a reversible capacity of 90.1 mAh g^−1^ at 5C with an energy density of 252.2 Wh kg^−1^ (Figures 5(b)–5(d), Figure S23, Figure S24a, and Table S8, Supporting Information). Besides, as shown in Figures 5(e)–5(g), Figure S24b, c, S25 (Supporting Information), the O3-NaNCMMT electrode not only reaches capacity retention of 86.2% with little polarization and stable energy efficiency as well as the mid-point voltage after 300 cycles at 1C but also shows better comprehensive performance.

## 3. Discussion

In summary, a stable Co-free O3-type NaNi_0.4_Cu_0.05_Mg_0.05_Mn_0.4_Ti_0.1_O_2_ cathode material with large-scale production for practical battery has been designed through partially substituting copper and magnesium into the nickel sites and titanium into the manganese sites. The rationally selected multielement chemical substitution could not only restrain the unfavorable multiphase transformation and suppress spontaneous Na extraction as well as enhance air stability but also optimize the structural reversibility and kinetics. All of the above account for the extraordinary performance of the O3-NaNCMMT electrode with a high specific capacity of 129.7 mAh g^−1^ at 0.2C, a superior rate capacity of 95.4 mAh g^−1^ at 10C, and a superlong cycle life over 1500 cycles even at 5 C after performance tests at various rates. The concept of the accurate multielement chemical substitution strategy shows great prospect to develop high-performance SIBs. This strategy is also expected to be applied to P2-type and NASICON-type cathode materials and their derivatives for the practical application of large-scale EESC systems.

## 4. Materials and Methods

### 4.1. Materials Synthesis

O3-type NaNi_0.4_Cu_0.05_Mg_0.05_Mn_0.4_Ti_0.1_O_2_ cathode material (hereafter denoted as O3-NaNCMMT) was synthesized by a high-temperature solid-state reaction process with a large-scale production line. The stoichiometric mixtures of Na_2_CO_3_ (99.5%; 5 mole percent access; Alfa Aesar), NiO (99.0%; Alfa Aesar), CuO (99.7%; Sinopharm), MgO (99%; Alfa Aesar), Mn_2_O_3_ (98.0%; Alfa Aesar), and TiO_2_ (anatase, 99.6%; Alfa Aesar) were ground and calcinated at 1000°C in air for 12 hours. The product was stored in an argon-filled glove box for further use (H_2_O and O_2_ < 0.1 parts per million).

### 4.2. Material Characterization

Powder XRD pattern was obtained by D8 Advance (Bruke, Germany) Diffractometer at a scan rate of 1°/min over the 10°-80°. *In situ* XRD test experiment was supported by a special Swagelok cell. Detailed morphological and crystalline structural information were obtained through both field-emission scanning electron microscopy (SU-8020, Hitachi Limited Corporation, Japan) and transmission electron microscopy (JEM 2100F, JEOL Limited Corporation, Japan). The atomic arrangement of cathode material was elucidated by scanning transmission electron microscopy (JEM-ARM200CF, JEOL, Tokyo, Japan).

### 4.3. Electrochemical Measurements

The electrochemical properties were evaluated in coin cells (CR2032) with Na disks and porous glass fiber as the counter electrode and separator, respectively. The electrolyte was a solution of 1 M NaClO_4_ in the mixed solvent of propylene carbonate and fluoroethylene carbonate (PC : FEC = 95 : 5, vol.%). The cathode film of the half-cell system was prepared by mixing the active material (70 wt%), Super P carbon (20 wt%), and polyvinylidene fluoride (10 wt%) binder in N-methyl-2-pyrrolidone (NMP) followed by casting slurry onto a clean Al foil and drying at 80°C in a vacuum oven overnight. Electrochemical measurements were performed on a Land BT2000 battery test system (1C = 120 mA g^−1^). The hard carbon anode of the full-cell system was prepared by a uniform mixture of 80 wt% active material, 10 wt% Super P carbon, and 10 wt% polyvinylidene difluoride (PVDF). The hard carbon anode was activated by an electrochemical presodiated process using the metal sodium foil as the counter electrode (1C = 300 mA g^−1^). The weight ratio of cathode and anode was balanced by referring to their reversible capacities. The electrochemical performance test of the full cell was also evaluated in the coin cell (CR2032), and the corresponding average loading of active material was about 2~3 mg cm^−2^. The current density was based on the mass of positive electrodes (1C = 120 mA g^−1^) with the voltage window of 1.9-3.9 V at room temperature.

## Data Availability

All data generated or analyzed during this study are included in this published article and its Supplementary Materials.

## Supplementary Materials

Supplementary Materials Figure S1: crystal structure of O3-NaNCMMT cathode material viewed along the (110) crystallographic direction. Figure S2: optical photograph of precursor concerning O3-NaNCMMT cathode material. Figure S3: (a–c) the colored patterns of HR-TEM image and FFT image as well as line profile of O3-NaNCMMT cathode material viewed along the (010) crystallographic direction. Figure S4: (a–c) the colored patterns of HR-TEM image and FFT image as well as line profile of O3-NaNCMMT cathode material viewed along the (001) crystallographic direction. Figure S5: (a–d) in-situ HEXRD patterns at different temperatures of formation process concerning O3-NaNCMMT cathode material. Figure S6: (a–f): in situ HEXRD patterns at different temperatures of thermal stability concerning O3-NaNCMMT cathode material. Figure S7: (a, b) intensity contour maps (bird's eye view) concerning the evolution of the characteristic diffraction peaks of in-situ HEXRD patterns of formation process and thermal stability concerning O3-NaNCMMT cathode material at different temperatures. Figure S8: (a, b) HAADF and ABF-STEM images and atomic model of O3-NaNCMMT cathode material viewed along the (010) crystallographic direction. Figure S9: (a–d) Typical FFT images and colored patterns of HAADF-STEM images concerning O3-NaNCMMT cathode material viewed along the (010) and (001) crystallographic directions, respectively. Figure S10: EELS spectra of Ti L-edges, O K-edge, Mn L-edges, and Ni L-edges with an increment of 2 nm per spectrum from the surface to the center. Figure S11: electrochemical performance of O3-NaNCMMT electrode in half-cell system. (a) Galvanostatic charge/discharge curves versus specific energy at 0.2C in the voltage range of 2.0-4.0 V. (b) Galvanostatic charge/discharge curves versus specific energy at various rates. (c) Rate performance as the rate comes back to 1C. (d) Linear fitting of the log (i) versus log (v) plots at different oxidation peaks. Figure S12: (a, b) the results of linear fitting concerning peak current versus square root of the scan rate and linear fitting of the log (i) versus log (v) plots at different oxidation peaks of O3-NaNCMMT electrode. Figure S13: Nyquist plots of EIS for O3-NaNCMMT electrode. Figure S14: galvanostatic charge/discharge curves versus specific capacity in different 100th, 200th, 300th, 400th, 500th, 600th, 700th, 800th, 900th, and 1000th cycles at 5C of O3-NaNCMMT electrode. Figure S15: electrochemical performance of O3-NaNCMMT electrode in half-cell system. (a) Cycling performance during 1000 cycles at 5C after performance tests at various rates. (b) Energy efficiency and mid-voltage during 1000 cycles at 5C after performance tests at various rates. (c) Cycling performance during 1500 cycles at 5C after performance tests at various rates. Figure S16: the discharge specific capacity retentions of O3-NaNCMMT electrode are selected every 100 cycles during 1000 cycles at 5C after performance tests at various rates in a half-cell system. Figure S17: (a, b) linear fitting concerning peak current versus square root of the scan rate and linear fitting of the log (i) versus log (v) plots at different oxidation peaks of O3-NaNCMMT electrode (exposing cathode materials to air for three days). Figure S18: (a, b) the results of linear fitting concerning peak current versus square root of the scan rate and linear fitting of the log (i) versus log (v) plots at different oxidation peaks of O3-NaNCMMT electrode (exposing cathode materials to air for three days). Figure S19: In situ XRD patterns during charge/discharge process at 0.1C in the voltage range of 2.0-4.0 V. Black asterisks represent peaks from Al window. Figure S20: crystallographic parameters change of the O3-NaNCMMT cathode material before and after Na extraction refined by the Rietveld method. Figure S21: (a, b) galvanostatic charge/discharge curves versus specific capacity and specific energy of hard carbon anode at different cycles (presodiated by an electrochemical process) at 0.1C. Figure S22: galvanostatic charge/discharge curves versus specific energy at 0.2C in the voltage range of 1.9-3.9 V in full-cell system. Figure S23: galvanostatic charge/discharge curves versus specific capacity in different 1st at 0.1C and 2nd, 3rd at 0.2C of O3-NaNCMMT electrode in full-cell system. Figure S24: (a) galvanostatic charge/discharge curves versus specific energy at various rates in the voltage range of 1.9-3.9 V. (b) Galvanostatic charge/discharge curves versus specific capacity in different 20th, 50th, 80th, 100th, 200th, and 300th cycles at 1C. (c) Energy efficiency and midvoltage during 300 cycles at 1C in full-cell system. Figure S25: the discharge specific capacity retentions of O3-NaNCMMT electrode are selected every 50 cycles during 300 cycles at 1C in full-cell system. Table S1: crystallographic parameters of the O3-NaNCMMT cathode material refined by the Rietveld method. Table S2: atomic site occupations of the O3-NaNCMMT cathode material refined by the Rietveld method. Table S3: ICP-MS result of the O3-NaNCMMT cathode material. Table S4: specific parameters of electrochemical performance at different rates concerning O3-NaNCMMT electrode in half-cell system. Table S5: summary of the CV results obtained at different scan rates concerning the O3-NaNCMMT cathode material. Table S6: summary of comprehensive performance for various chemical element substituted O3-NaNi_0.5_Mn_0.5_O_2_ cathode materials. Table S7: summary of the CV results obtained at different scan rates after exposing O3-NaNCMMT cathode material to air for three days. Table S8: specific parameters of electrochemical performance at different rates concerning O3-NaNCMMT electrode in full-cell system. (Supplementary Materials)
