# Supplementary material for: Large-Scale Synthesis of the Stable Co-Free Layered Oxide Cathode by the Synergetic Contribution of Multielement Chemical Substitution for Practical Sodium-Ion Battery
Source: Research (Wash D C). 2020 Oct 19;2020:1469301. doi: 10.34133/2020/1469301 (PMC7592082; doi:10.34133/2020/1469301)

Supporting information for

Title

Large-Scale Synthesis of the Stable Co-Free Layered Oxide Cathode by the Synergetic Contribution of Multielement Chemical Substitution for Practical Sodium-Ion Battery

**Authors**

Yao Xiao^1,2^, Tao Wang^3^, Yan-Fang Zhu^2^, Hai-Yan Hu^1^, Shuang-Jie Tan^1,4^, Shi Li^2^, Peng-Fei Wang^1^, Wei Zhang^1^, Yu-Bin Niu^1^, En-Hui Wang^1^, Yu-Jie Guo^1,4^, Xinan Yang^5^, Lin Liu^1^, Yu-Mei Liu^2^, Hongliang Li^3^, Xiao-Dong Guo^2^*, Ya-Xia Yin^1,4^*, and Yu-Guo Guo^1,4^*

**Affiliations**

*^1^CAS Key Laboratory of Molecular Nanostructure and Nanotechnology, CAS Research/Education Center for Excellence in Molecular Sciences, Beijing National Laboratory for Molecular Sciences (BNLMS), Institute of Chemistry, Chinese Academy of Sciences (CAS), Beijing 100190, P. R. China*

*^2^School of Chemical Engineering, Sichuan University, Chengdu 610065, P. R. China*

*^3^Institute of Materials for Energy and Environment, College of Materials Science and Engineering, Qingdao University, Qingdao 266071, P. R. China*

*^4^University of Chinese Academy of Sciences, Beijing 100049, P. R. China*

*^5^Beijing National Laboratory for Condensed Matter Physics, Institute of Physics, Chinese Academy of Sciences (CAS), Beijing 100190, P. R. China*

Correspondence should be addressed to Xiao-Dong Guo; xiaodong2009@163.com, Ya-Xia Yin; yxyin@iccas.ac.cn, and Yu-Guo Guo; ygguo@iccas.ac.cn


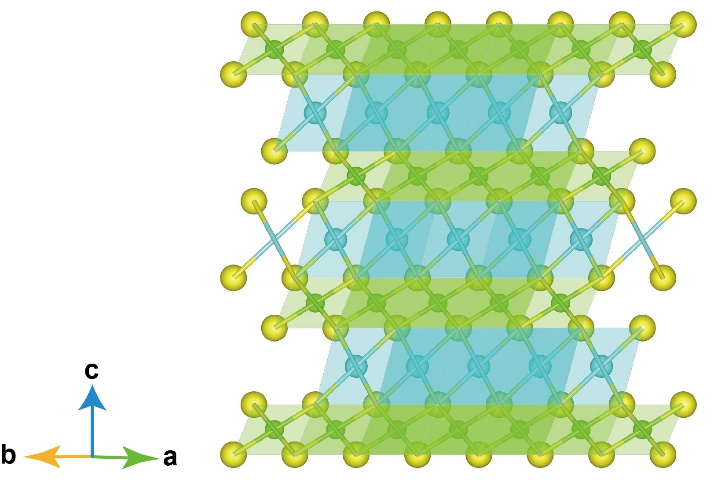


**Figure S1.** Crystal structure of O3-NaNCMMT cathode material viewed along the [110] crystallographic direction.


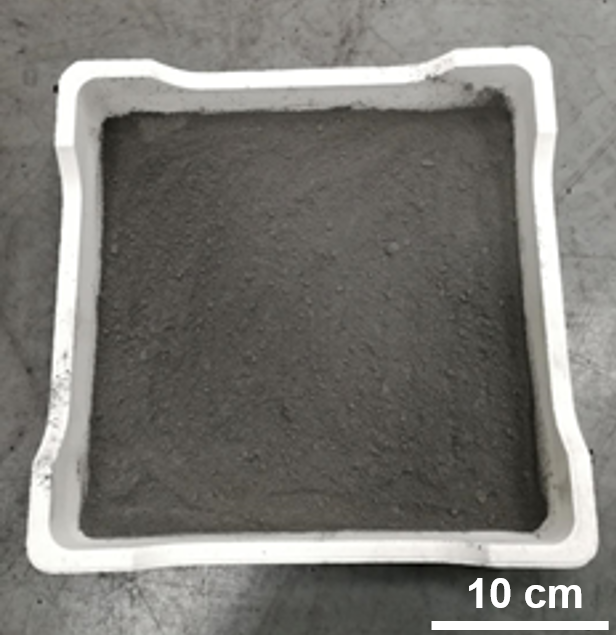


**Figure S2.** Optical photograph of precursor concerning O3-NaNCMMT cathode material.


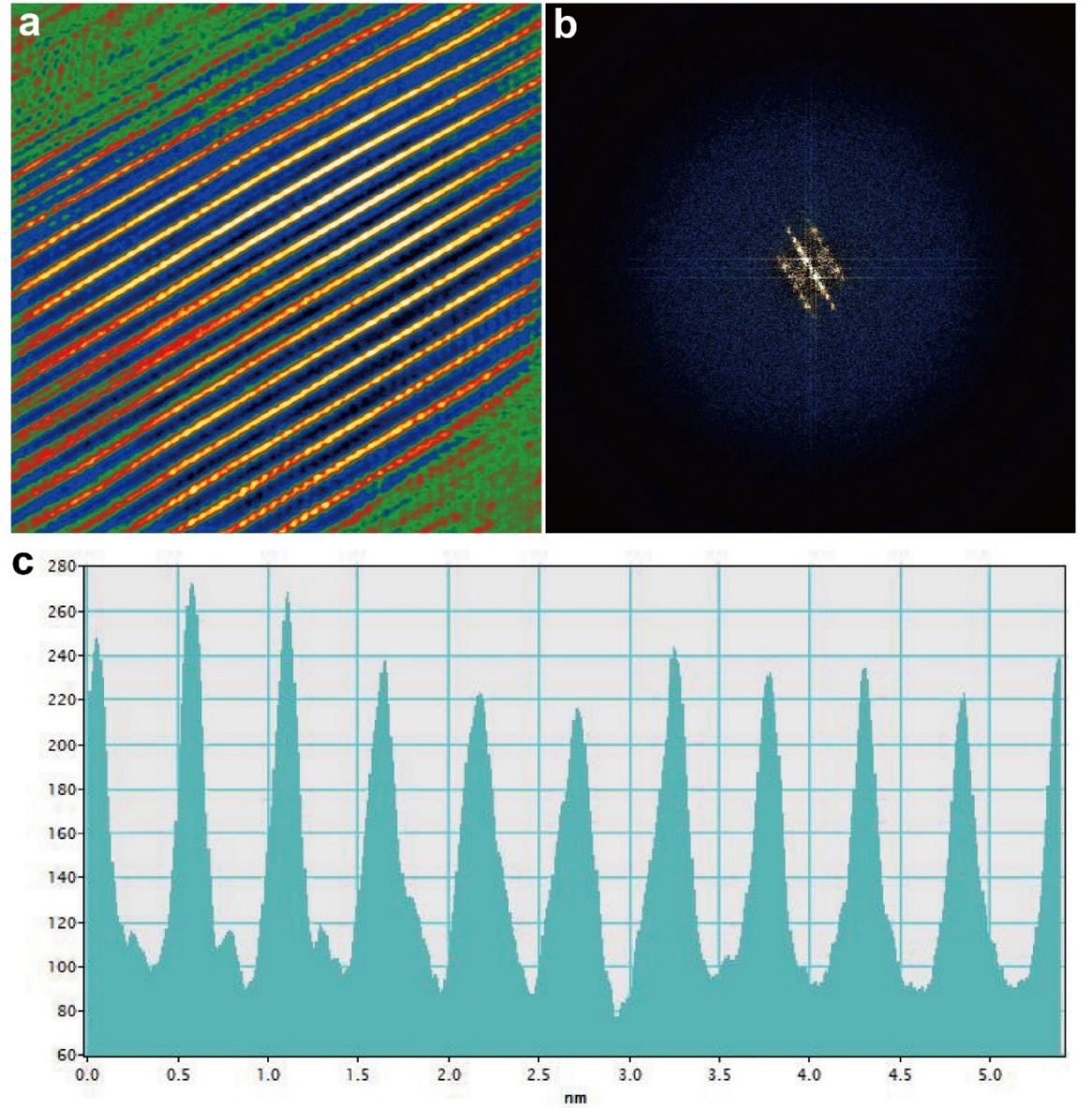


**Figure S3.** (a−c) The colored patterns of HR-TEM image and FFT image as well as line profile of O3-NaNCMMT cathode material viewed along the [010] crystallographic direction.

**
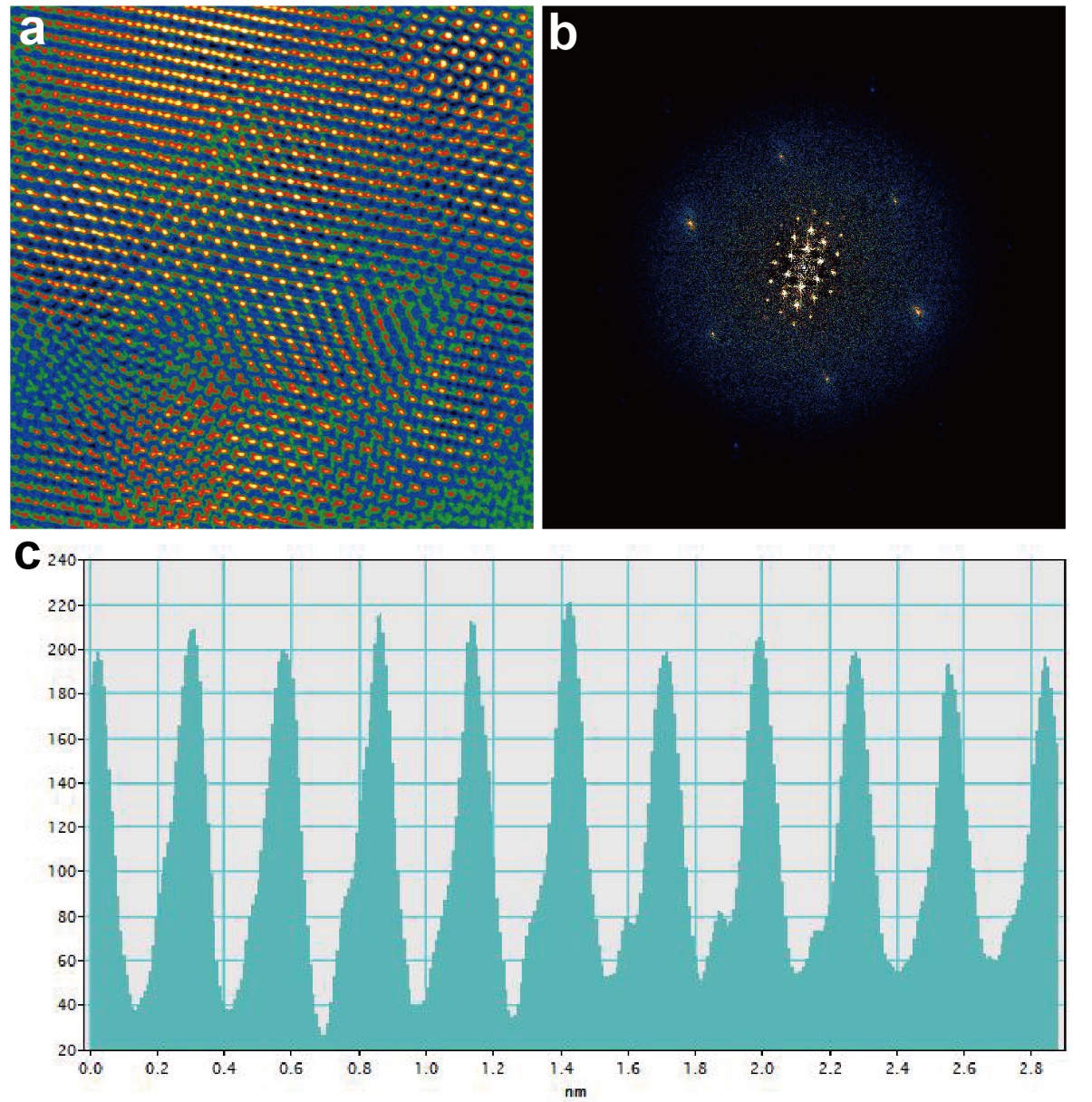
**

**Figure S4.** (a−c) The colored patterns of HR-TEM image and FFT image as well as line profile of O3-NaNCMMT cathode material viewed along the [001] crystallographic direction.

**
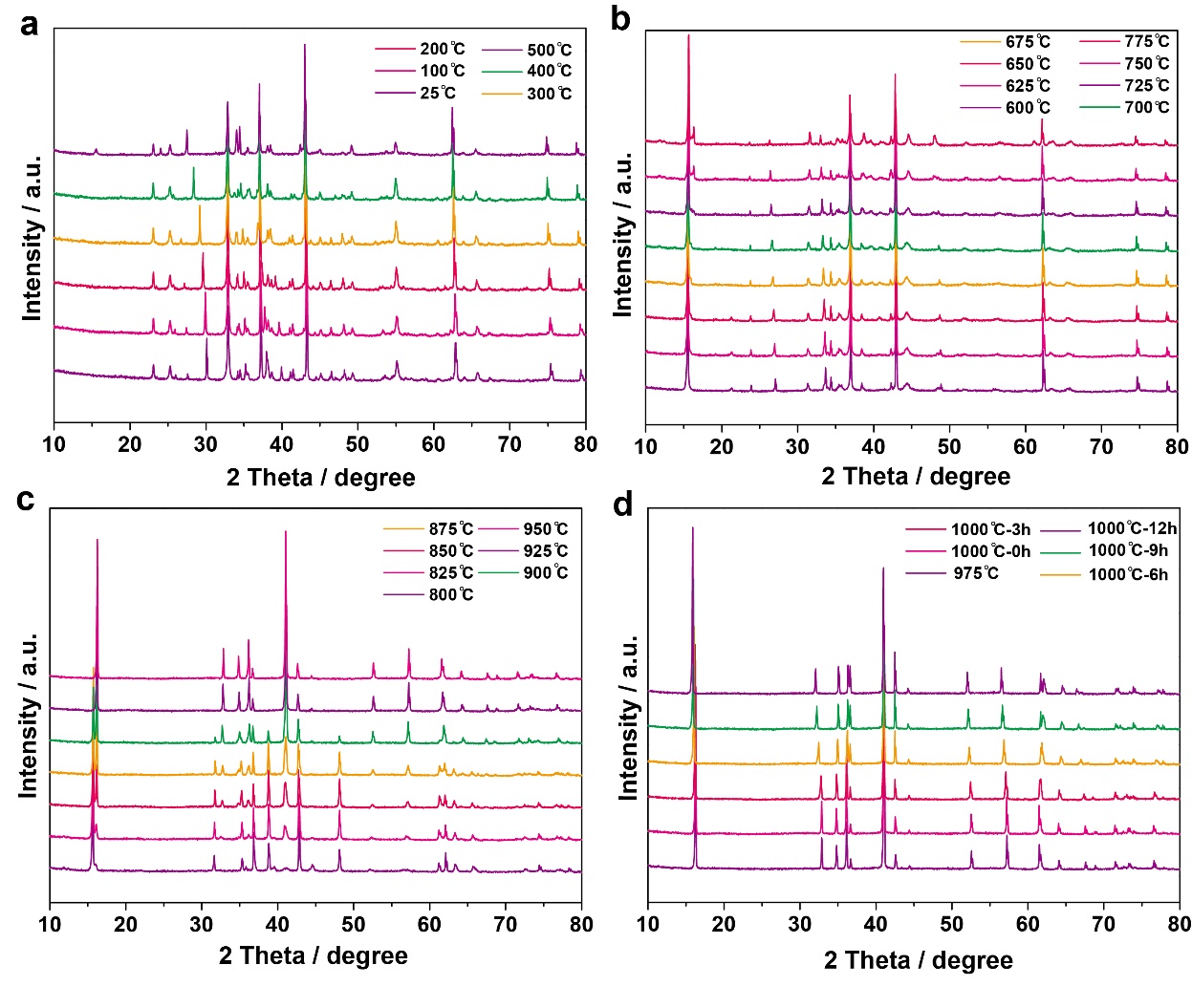
**

**Figure S5.** (a−d) *In-situ* HEXRD patterns at different temperatures of formation process concerning O3-NaNCMMT cathode material.

The formation process is clearly illustrated by *in-situ* HEXRD in air atmosphere from room temperature to 900 °C, during which the characteristic peaks of transition metal oxide can be observed from 25 °C to 400 °C. (003) Characteristic peak of the *α*-NaFeO_2_ layered structure begins to form when the temperature rises to 500 °C, which is followed by the gradually stronger intensity of (003) characteristic peak as the temperature continues to increase to 600 °C. Meanwhile, other diffraction peaks also progressively disappear from 600 °C to 800 °C, while O3-type layered structure observed via its main characteristic peaks is generated when the temperature increases to 950°C, which still shows stability and maintains high crystallinity as the temperature rises to 1000 °C.


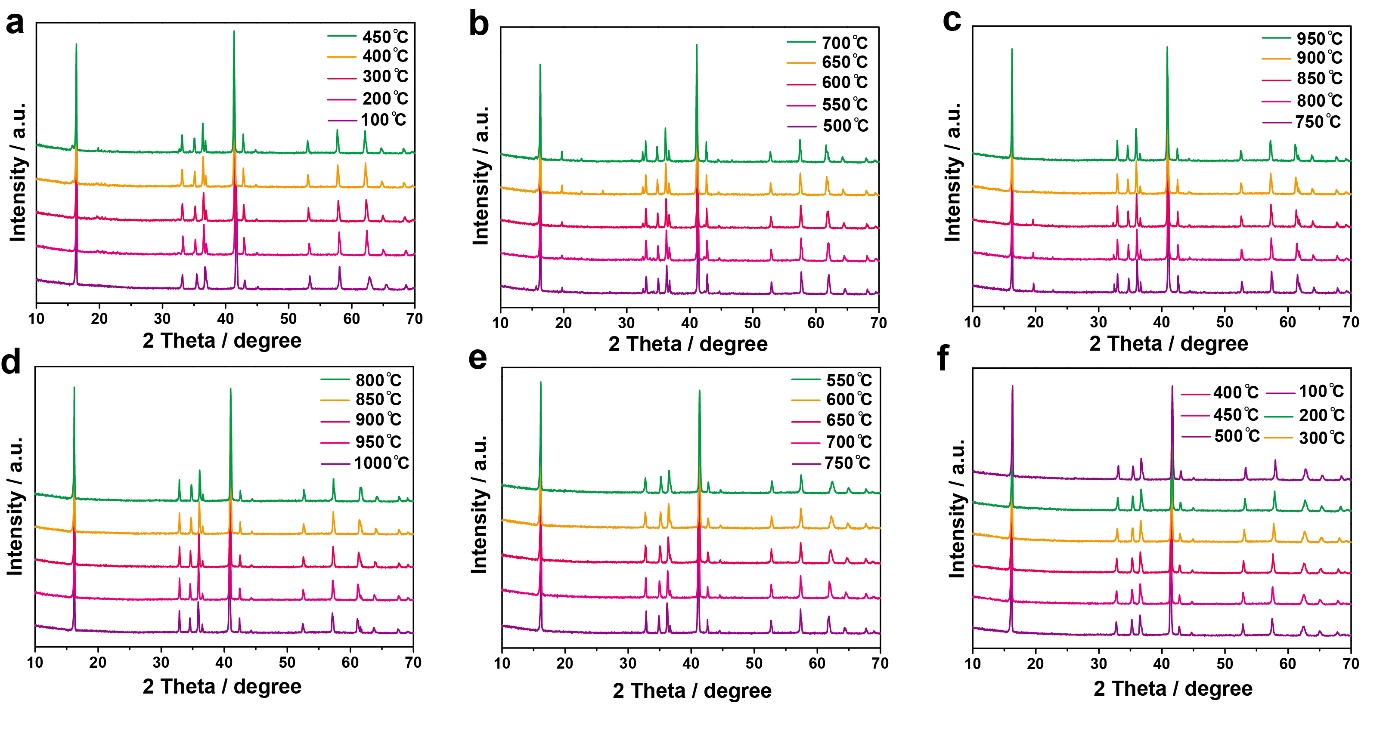
**Figure S6. (**a−f) *In-situ* HEXRD patterns at different temperatures of thermal stability concerning O3-NaNCMMT cathode material.

**
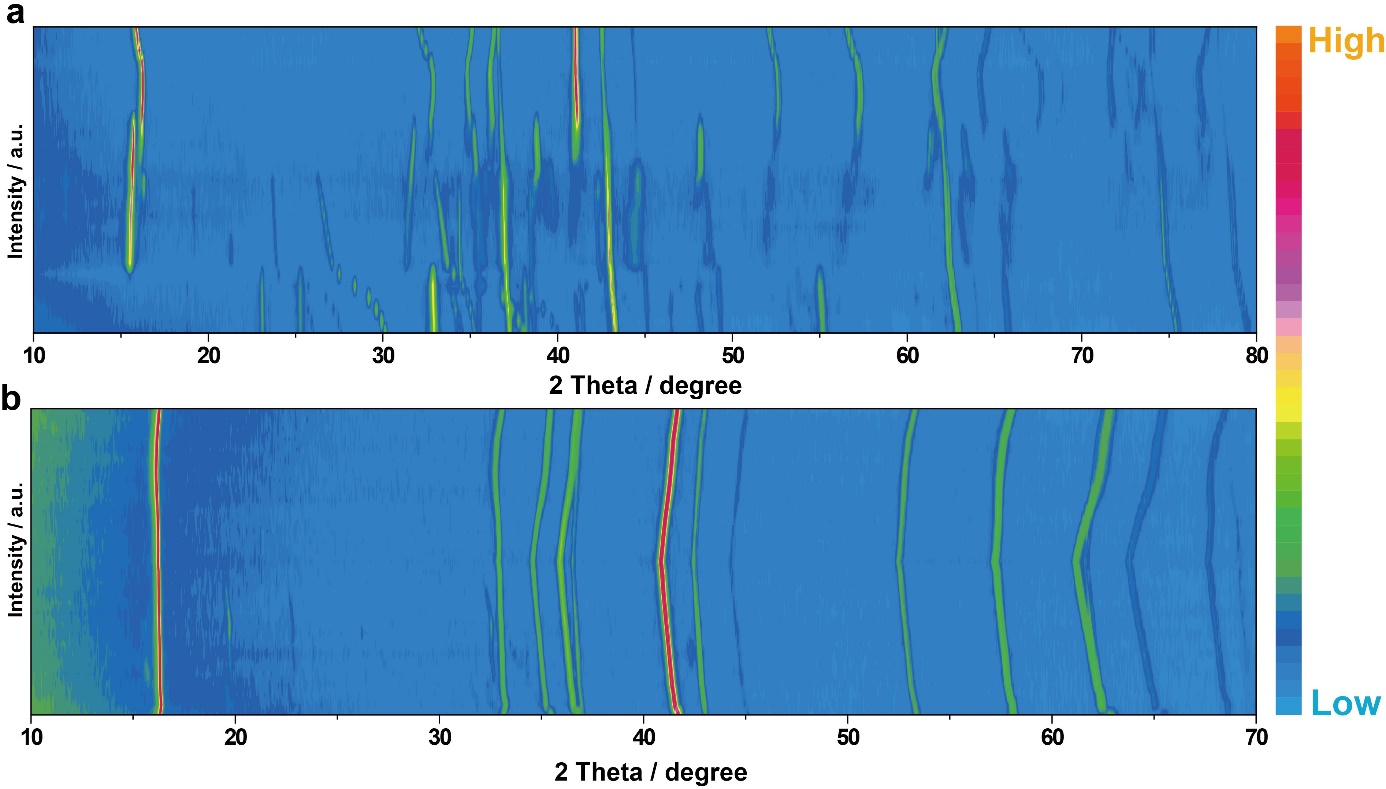
**

**Figure S7.** (a, b) Intensity contour maps (bird’s eye view) concerning the evolution of the characteristic diffraction peaks of *in-situ* HEXRD patterns of formation process and thermal stability concerning O3-NaNCMMT cathode material at different temperatures.


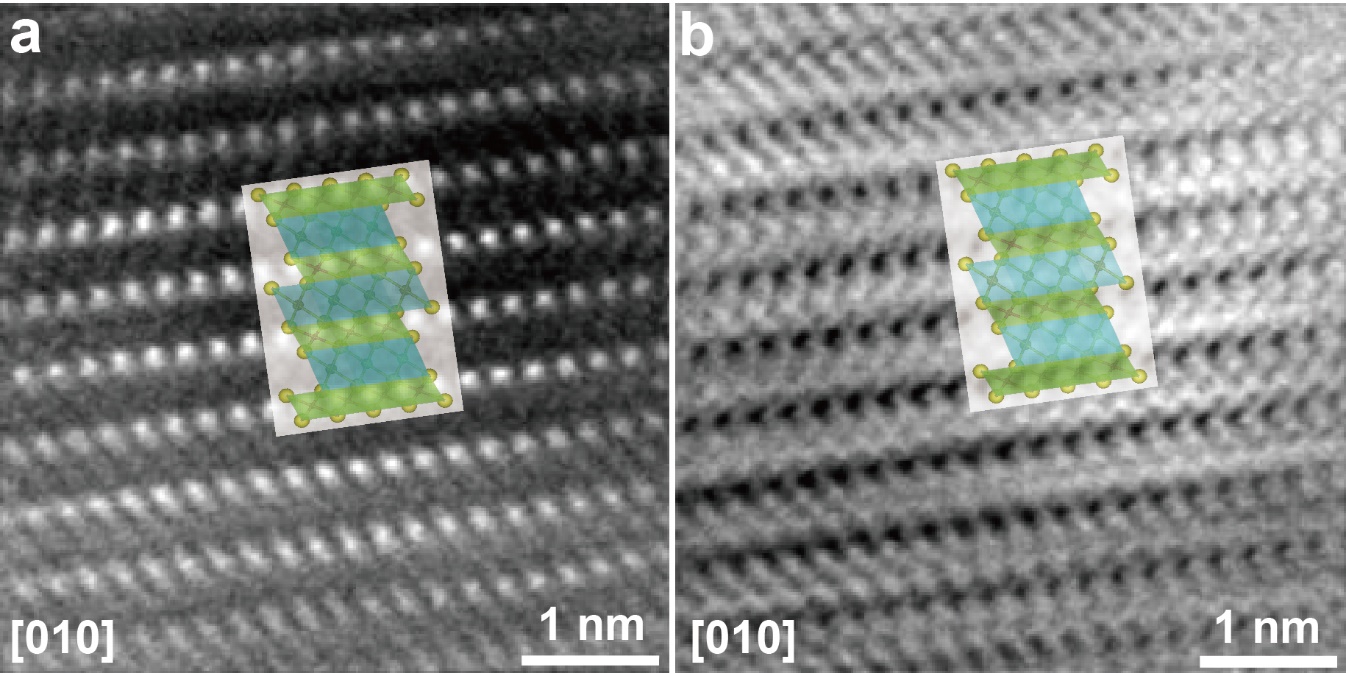


**Figure S8.** (a, b) HAADF and ABF-STEM images and atomic model of O3-NaNCMMT cathode material viewed along the [010] crystallographic direction.

**
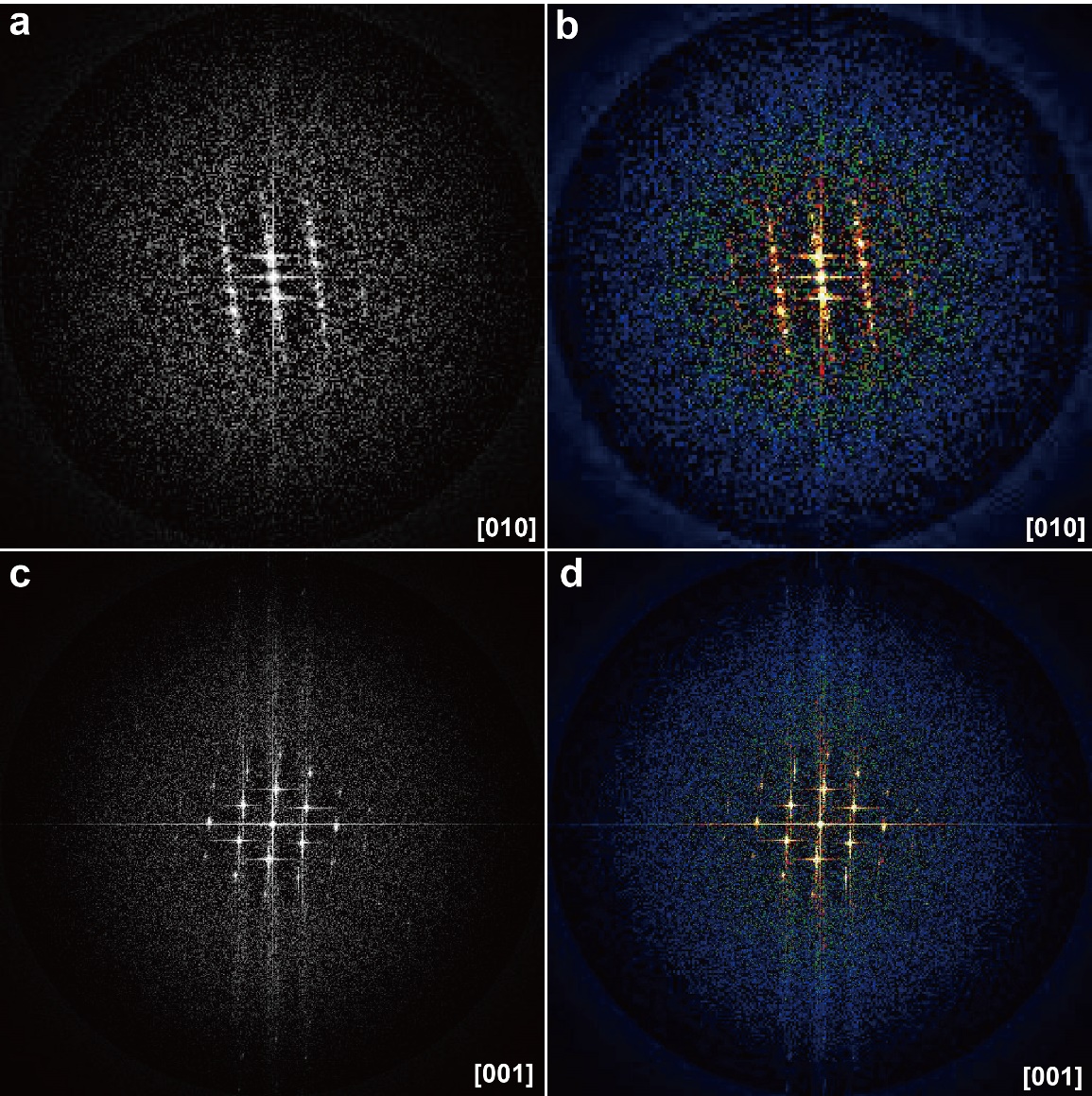
**

**Figure S9.** (a−d) Typical FFT images and colored patterns of HAADF-STEM images concerning O3-NaNCMMT cathode material viewed along the [010] and [001] crystallographic directions, respectively.


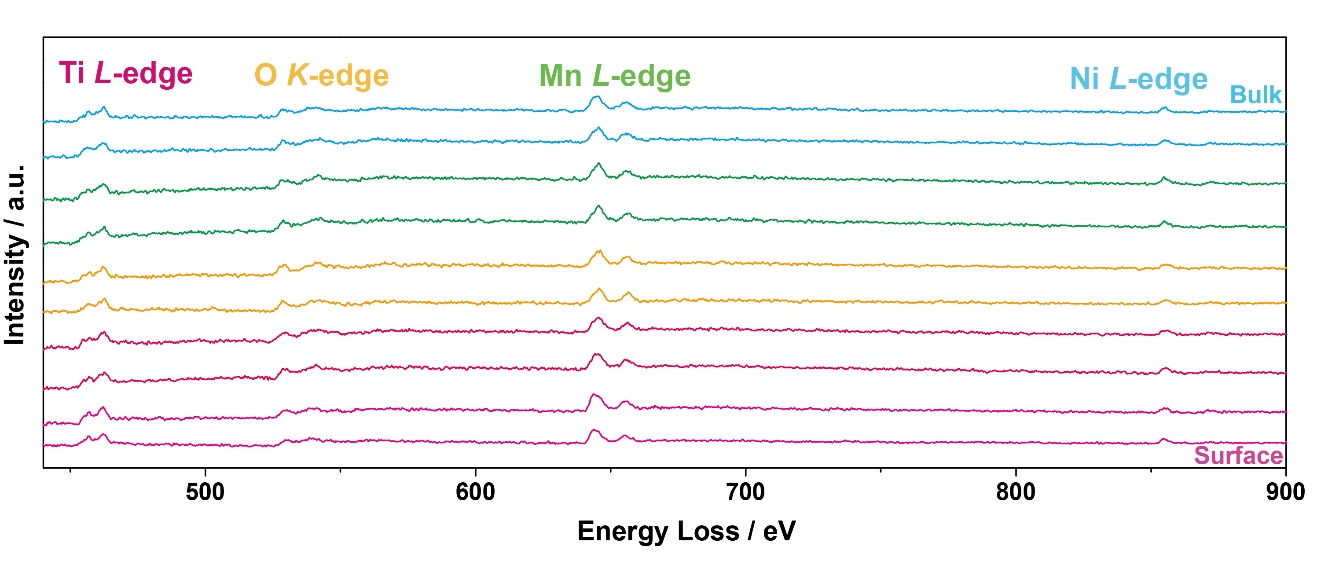


**Figure S10.** EELS spectra of Ti *L*-edges, O *K*-edge, Mn *L*-edges, and Ni *L*-edges with an increment of 2 nm per spectrum from the surface to the center.

**
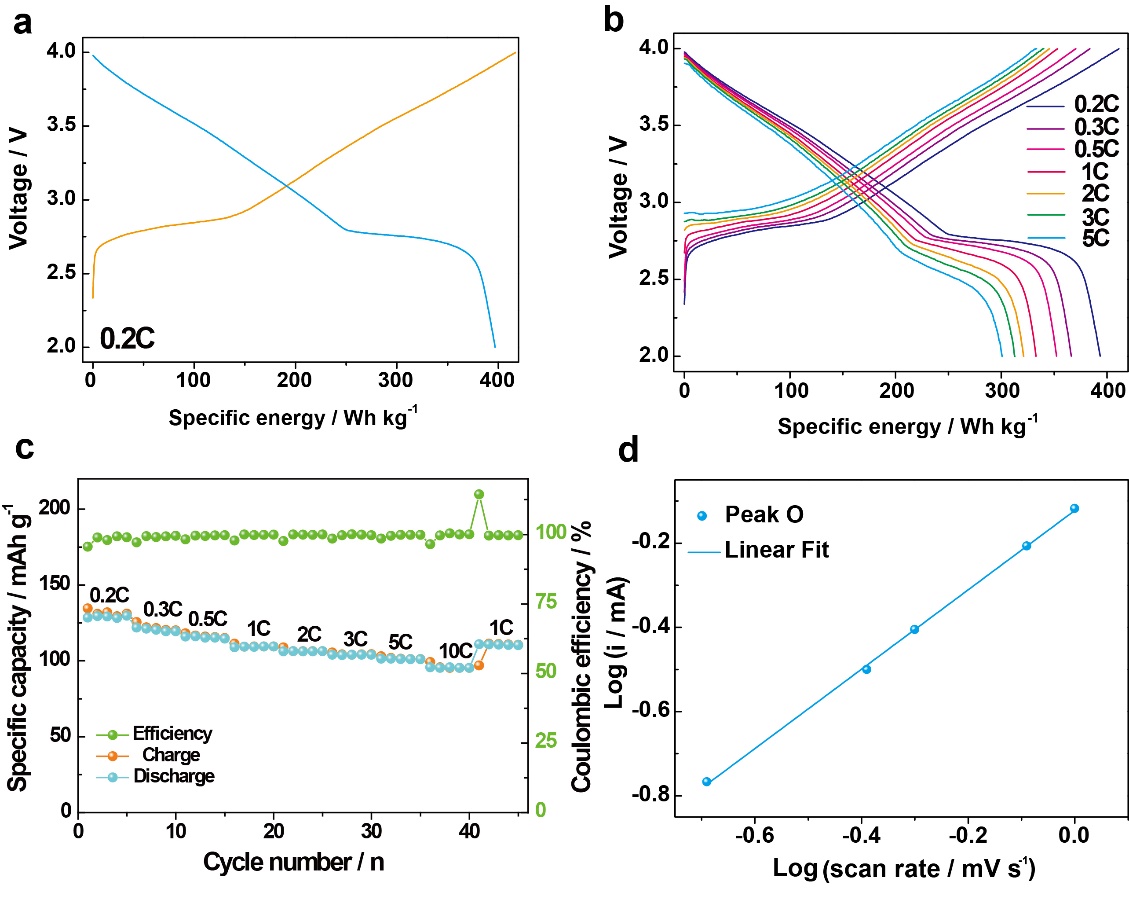
**

**Figure S11.** Electrochemical performance of O3-NaNCMMT electrode in half-cell system. (a) Galvanostatic charge/discharge curves versus specific energy at 0.2C in the voltage range of 2.0-4.0 V. (b) Galvanostatic charge/discharge curves versus specific energy at various rates. (c) Rate performance as the rate comes back to 1C. (d) Linear fitting of the log (*i*) versus log (*v*) plots at different oxidation peaks.


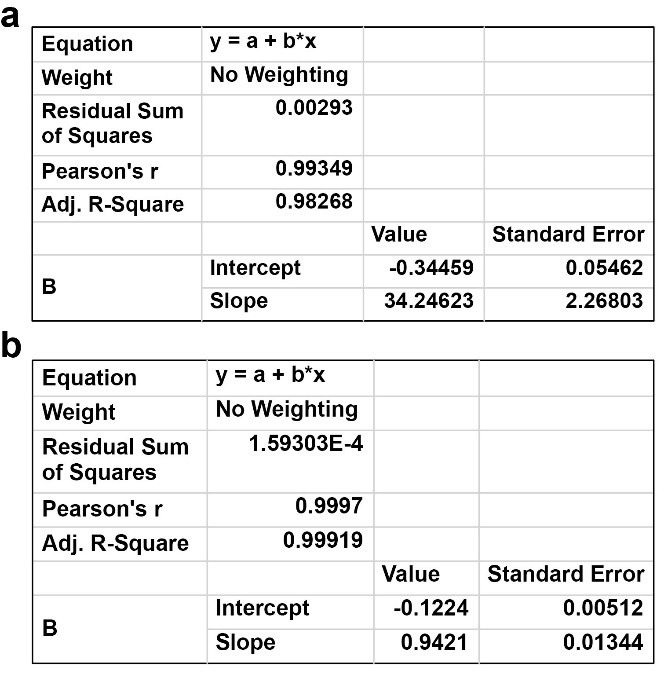


**Figure S12. (**a, b) The results of linear fitting concerning peak current versus square root of the scan rate and linear fitting of the log (*i*) versus log (*v*) plots at different oxidation peaks of O3-NaNCMMT electrode.

The Na^+^ apparent diffusion coefficient in O3-NaNCMMT cathode material was calculated according to the Randles-Sevcik Equation:

*I_p_ =* 0.4463*n^3/2^F^3/2^CSR*^–^*^1/2^T*^–^*^1/2^D_cv_^1/2^v^1/2^*

where *v* (V s^–1^) is the scan rate, *D_CV_* (cm^2^ s^–1^) is the apparent diffusion coefficient, *T* (K) is the absolute temperature, *R* is the gas constant (8.314 J mol^−1^ K^−1^), *S* (cm^2^) is the area of the electrode, *C* (mol cm^–3^) is the inserted Na^+^ concentration in cathode material, *F* is Faraday constant (96485 C mol^–1^), n is the number of moles of electrons transferred in the reaction, and *I_p_* (A) is peak current. Accordingly, the estimated diffusion coefficient is 1.578×10^–11^ cm^2^ s^–1^. Besides, the peak current obeys a power-law relationship with the scan rate based on the equation *i* = *av^b^*. The b value of 0.5 means that the electrode reaction is dominated by a diffusion-controlled process, and the b value of 1.0 means a capacitive controlled process. Here, the b value determined by the slope of log(*i*) versus log(*v*) at different oxidation peaks is 0.9421, suggesting a mixed-contribution of diffusion and capacitive controlled process.

**
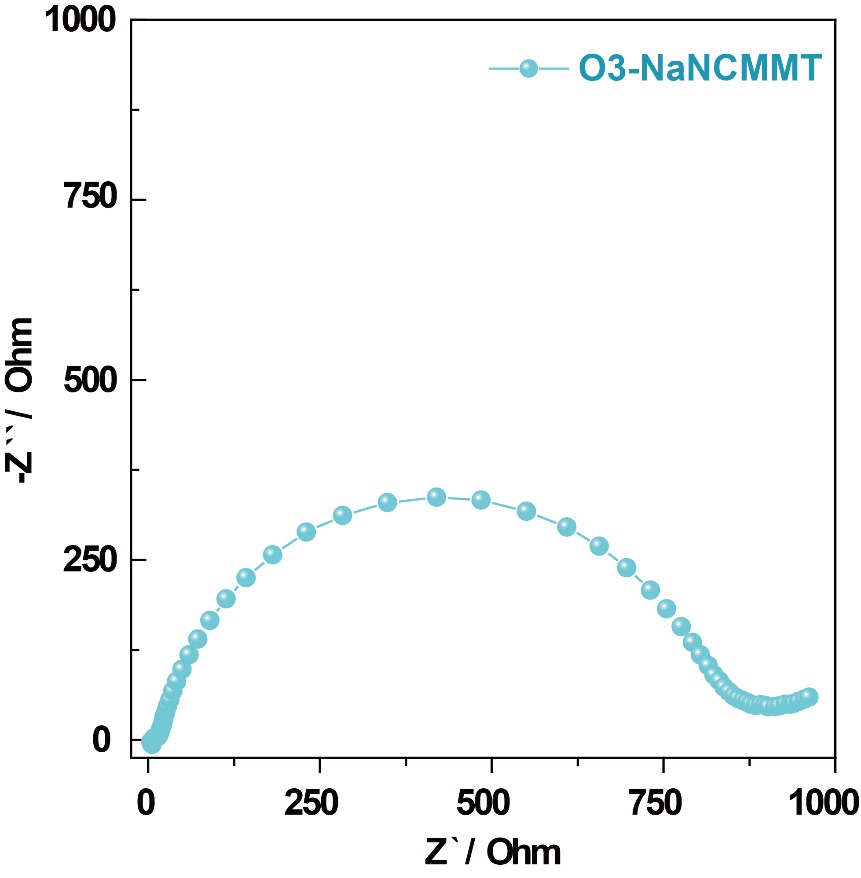
**

**Figure S13.** Nyquist plot of EIS for O3-NaNCMMT electrode.

The EIS curve for O3-NaNCMMT electrode consists of a depressed semicircle in the high-to-medium frequency and a straight line in the low-frequency region. The depressed semicircle in the high-to-medium frequency regions refers to the charge transfer resistance (*R*_ct_), and the oblique line in the low frequency reflects the Warburg impedance (*Z*_w_), which relates to Na^+^ diffusion in the bulk of the electrode. A relatively lower charge transfer resistance (*R*_ct_) at the electrode/electrolyte interface and faster Na^+^ diffusion associated with Warburg impedance (*Z*_w_) are obtained for O3-NaNCMMT, suggesting that the multielement chemical substitution strategy facilitates Na^+^ migration.

**
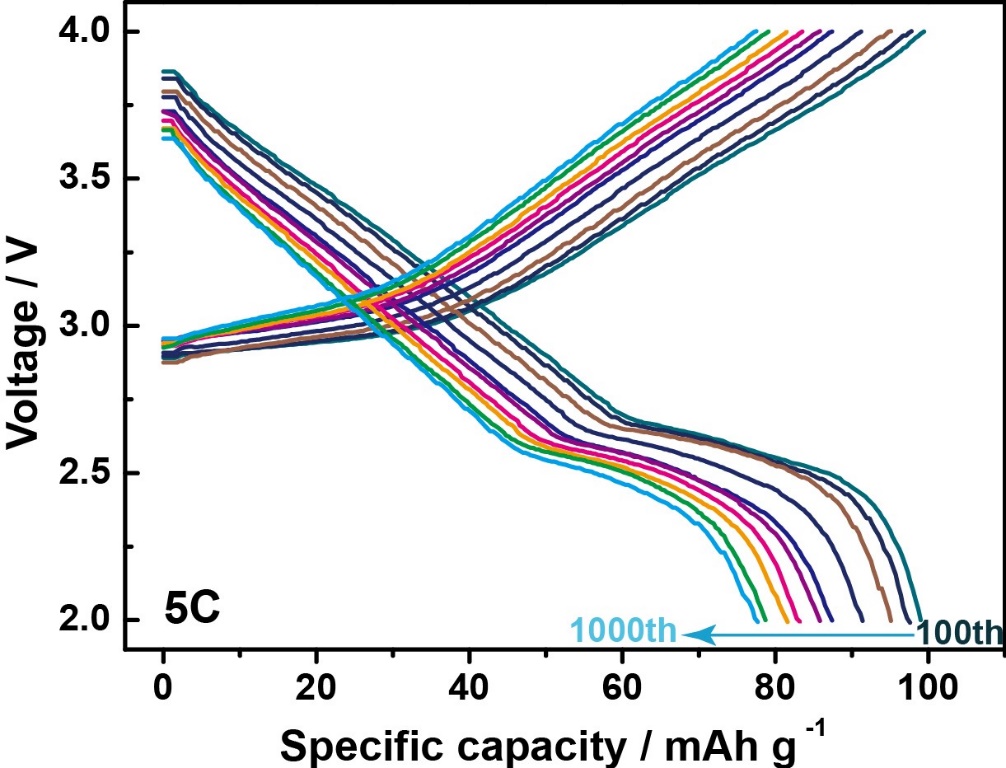
**

**Figure S14.** Galvanostatic charge/discharge curves versus specific capacity in different 100th, 200th, 300th, 400th, 500th, 600th, 700th, 800th, 900th, and 1000th cycles at 5C of O3-NaNCMMT electrode.

**
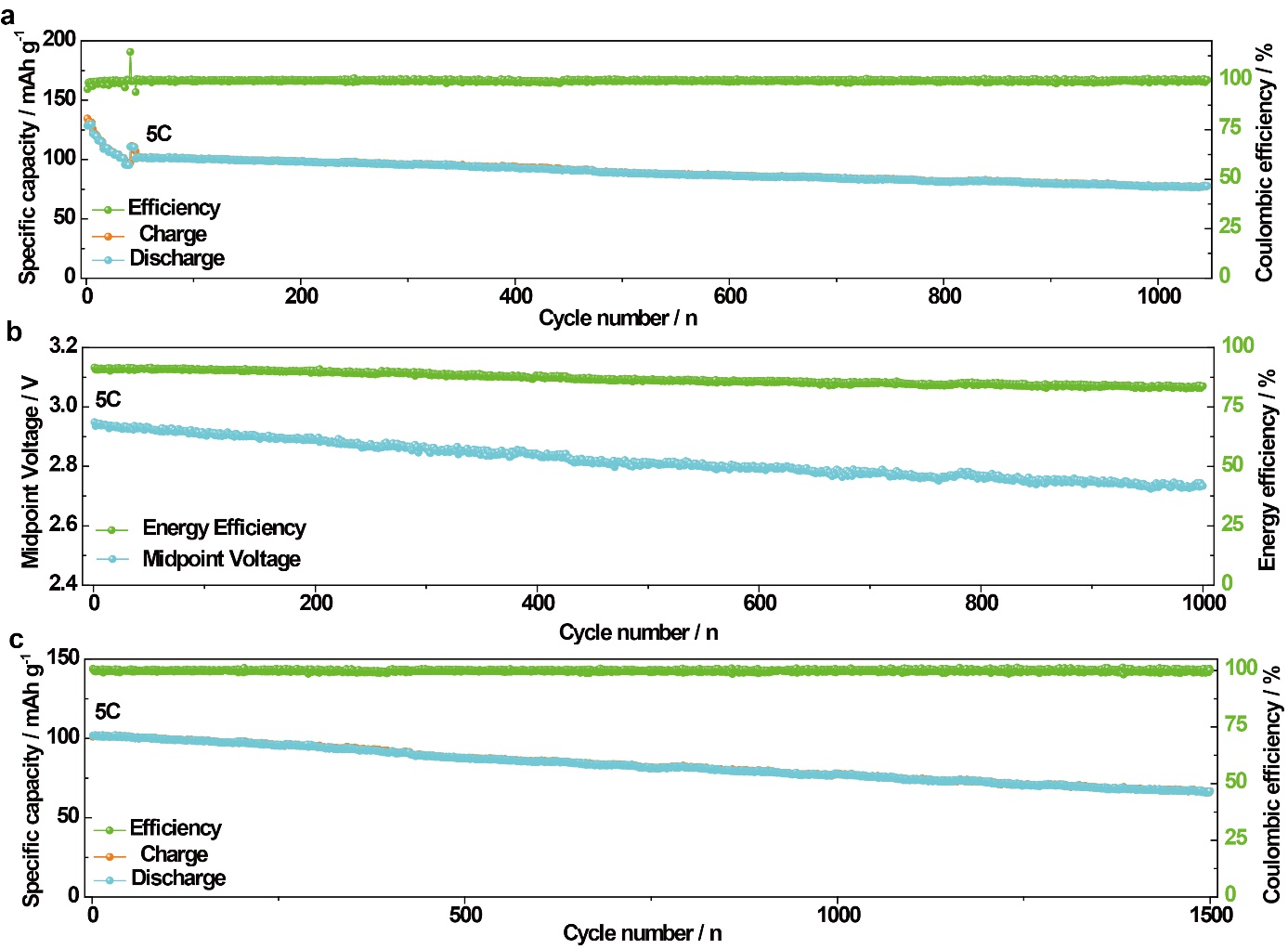
**

**Figure S15.** Electrochemical performance of O3-NaNCMMT electrode in half-cell system. (a) Cycling performance during 1000 cycles at 5C after performance tests at various rates. (b) Energy efficiency and mid-voltage during 1000 cycles at 5C after performance tests at various rates. (c) Cycling performance during 1500 cycles at 5C after performance tests at various rates.

**
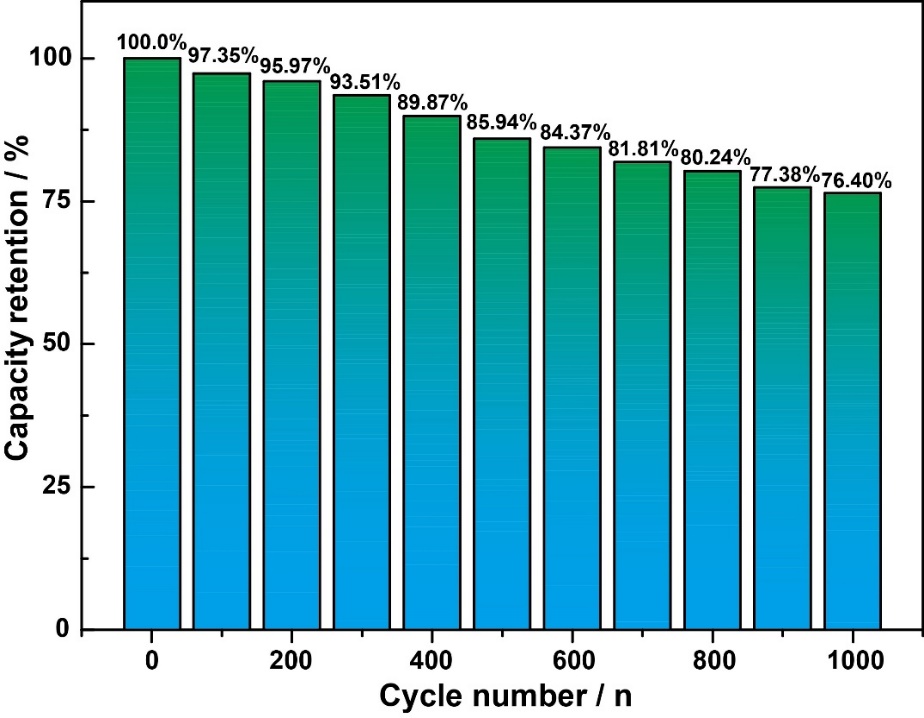
**

**Figure S16.** The discharge specific capacity retentions of O3-NaNCMMT electrode are selected every 100 cycles during 1000 cycles at 5C after performance tests at various rates in half-cell system.


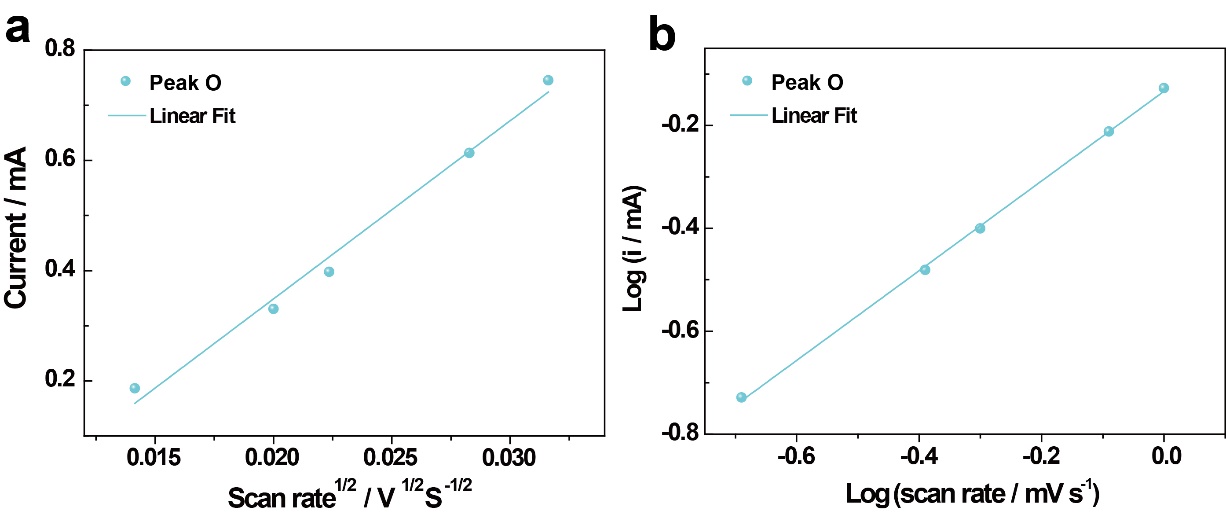


**Figure S17. (**a, b) Linear fitting concerning peak current versus square root of the scan rate and linear fitting of the log (*i*) versus log (*v*) plots at different oxidation peaks of O3-NaNCMMT electrode (exposing cathode materials to air for three days).

**
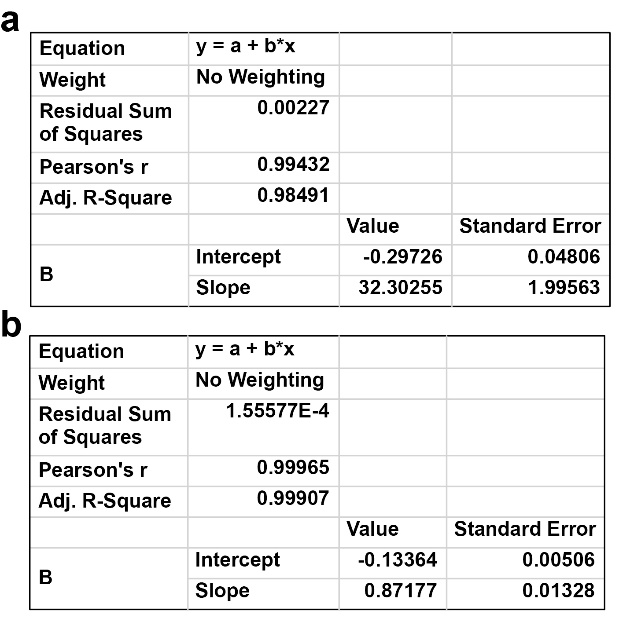
**

**Figure S18. (**a, b) The results of linear fitting concerning peak current versus square root of the scan rate and linear fitting of the log (*i*) versus log (*v*) plots at different oxidation peaks of O3-NaNCMMT electrode (exposing cathode materials to air for three days).

According to the two equations in the Figure S10, after exposing O3-NaNCMMT cathode material to air for 3 days, the diffusion coefficient is 1.404×10^–11^ cm^2^ s^–1^, and the b value is 0.8718.

**
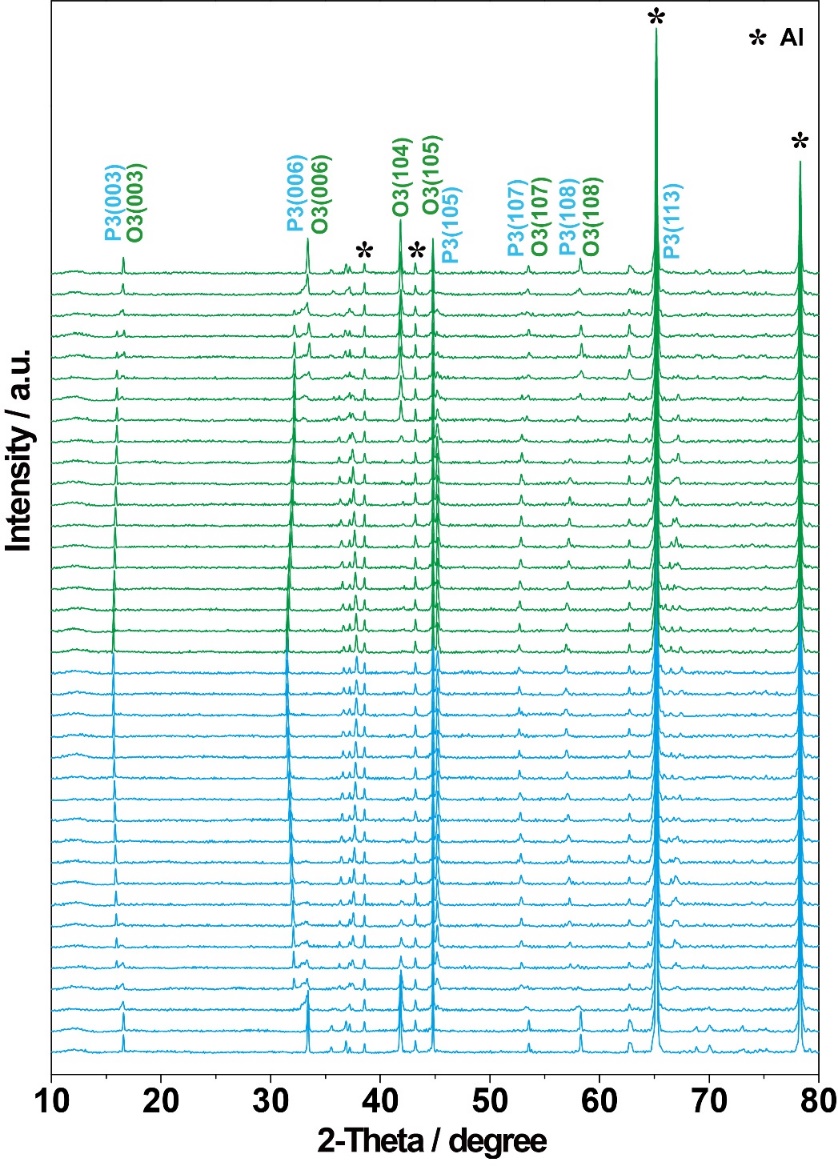
**

**Figure S19.** *In-situ* XRD patterns during charge/discharge process at 0.1C in the voltage range of 2.0-4.0 V. Black asterisks represent peaks from Al window.


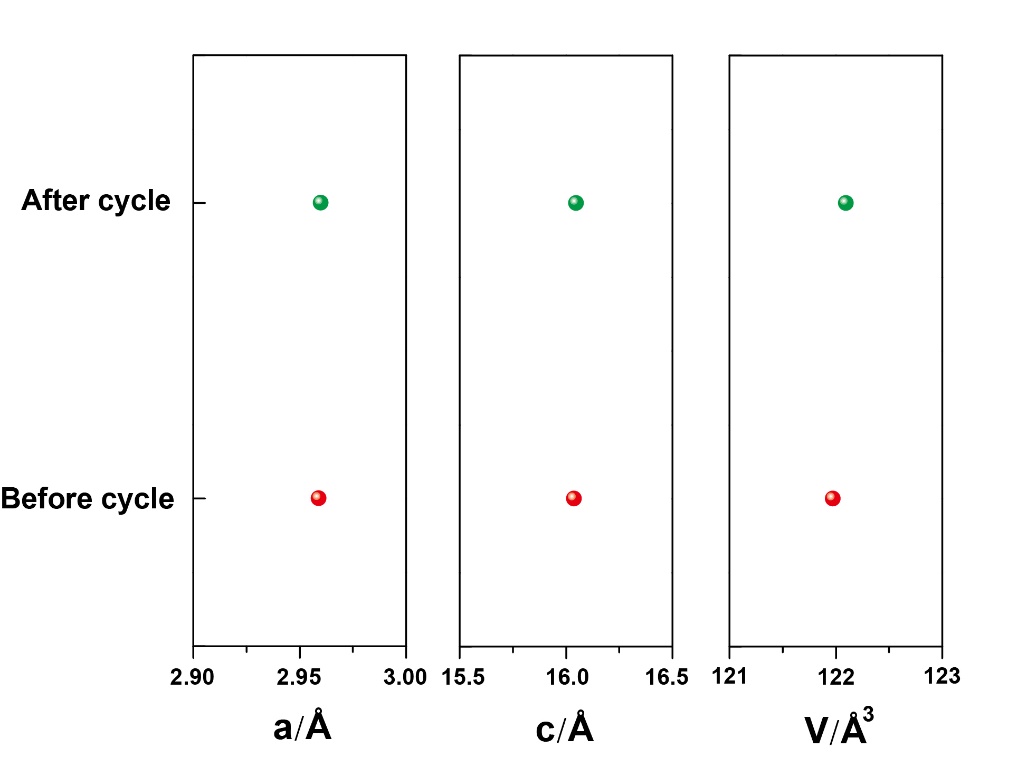


**Figure S20.** Crystallographic parameters change of the O3-NaNCMMT cathode material before and after Na extraction refined by the Rietveld method.

**
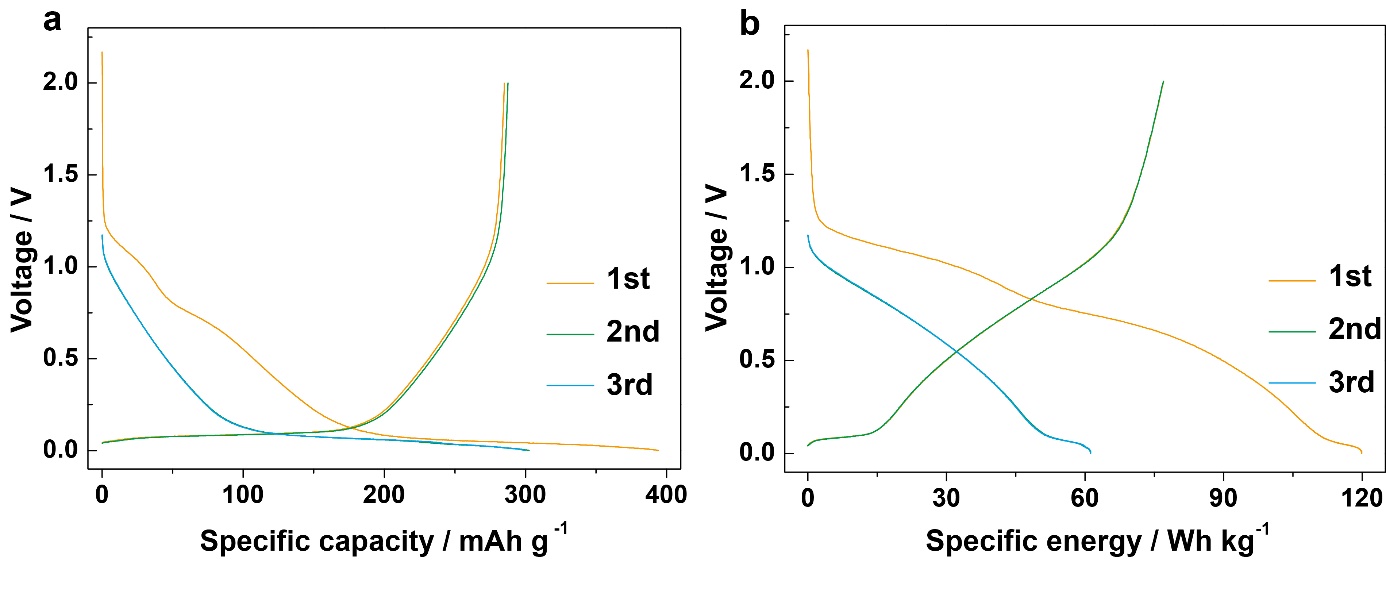
**

**Figure S21. (**a, b) Galvanostatic charge/discharge curves versus specific capacity and specific energy of hard carbon anode at different cycles (pre-sodiated by an electrochemical process) at 0.1C.

**
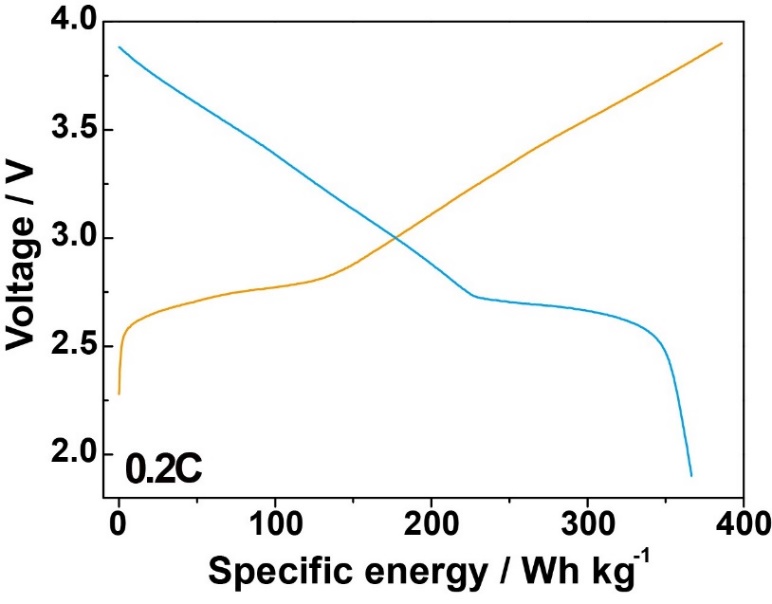
**

**Figure S22.** Galvanostatic charge/discharge curves versus specific energy at 0.2C in the voltage range of 1.9-3.9 V in full-cell system.


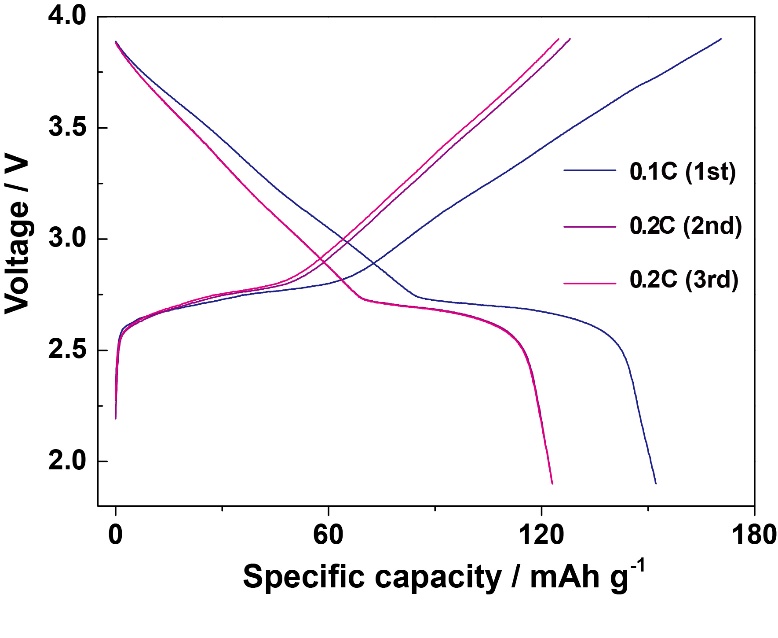


**Figure S23.** Galvanostatic charge/discharge curves versus specific capacity in different 1st at 0.1C and 2nd, 3rd at 0.2C of O3-NaNCMMT electrode in full-cell system.

**
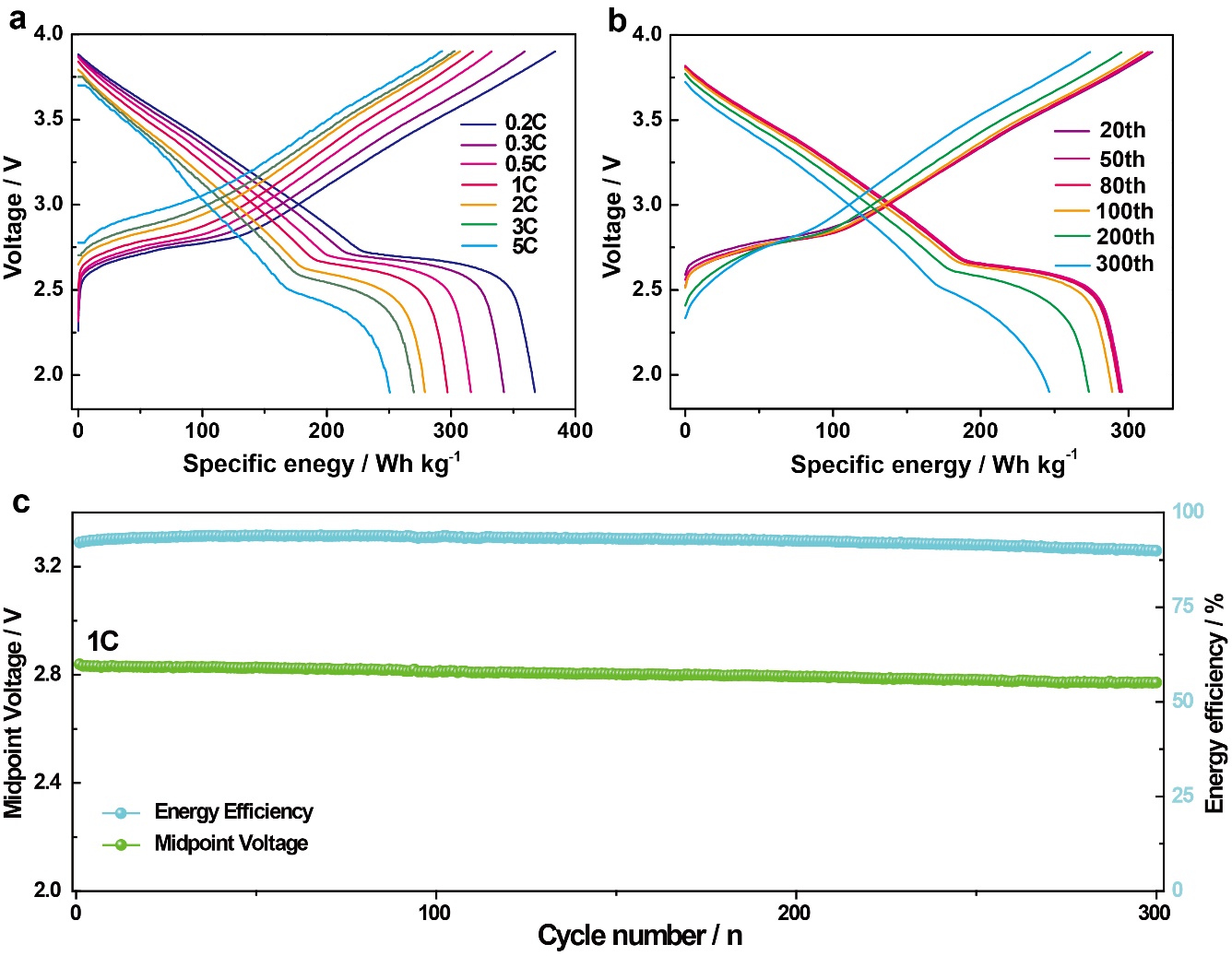
**

**Figure S24.** (a) Galvanostatic charge/discharge curves versus specific energy at various rates in the voltage range of 1.9-3.9 V. (b) Galvanostatic charge/discharge curves versus specific capacity in different 20th, 50th, 80th, 100th, 200th, and 300th cycles at 1C. (c) Energy efficiency and mid-voltage during 300 cycles at 1C in full-cell system.


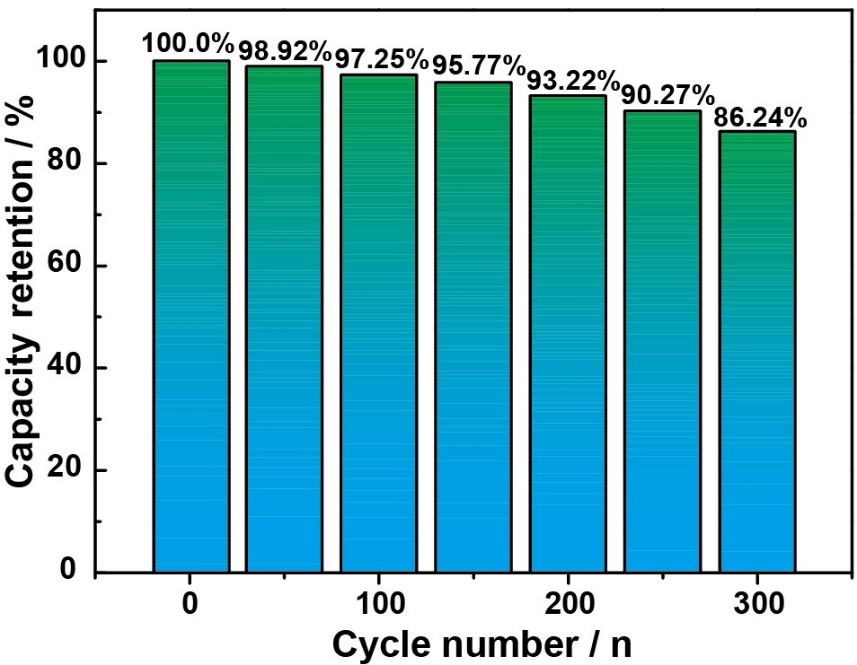


**Figure S25.** The discharge specific capacity retentions of O3-NaNCMMT electrode are selected every 50 cycles during 300 cycles at 1C in full-cell system.

**Table S1.** Crystallographic parameters of the O3-NaNCMMT cathode material refined by the Rietveld method.

**
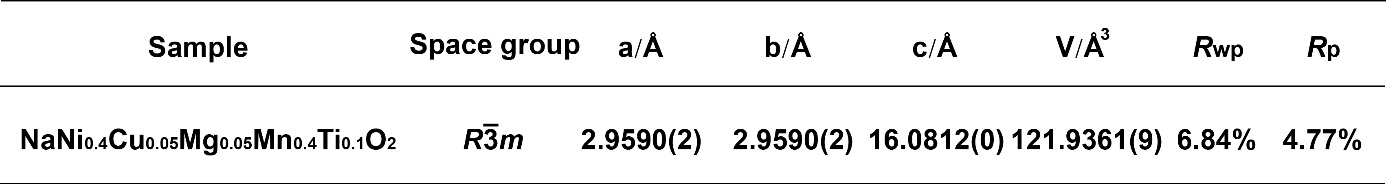
**

**Table S2.** Atomic site occupations of the O3-NaNCMMT cathode material refined by the Rietveld method.

**
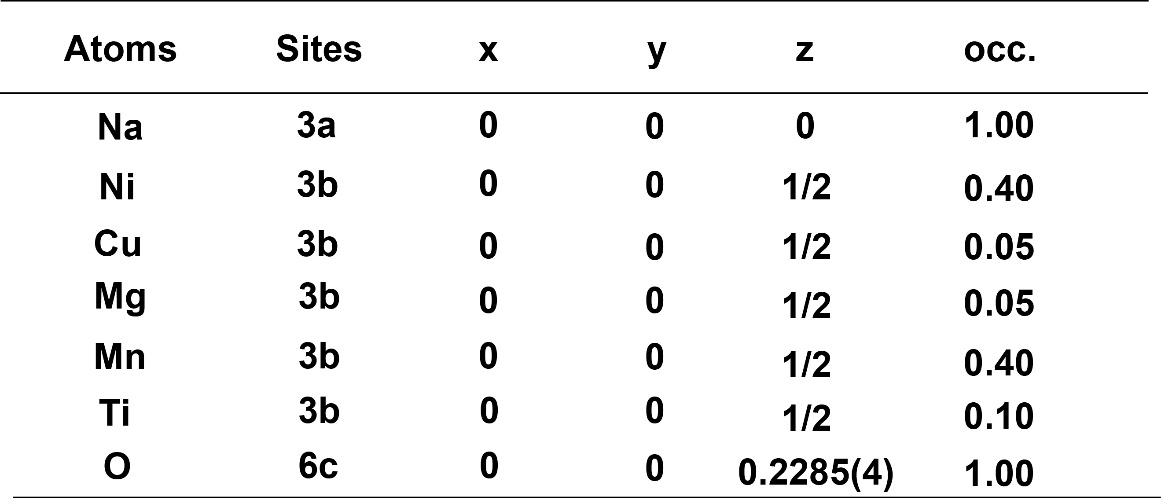
**

**Table S3.** ICP-MS result of the O3-NaNCMMT cathode material.


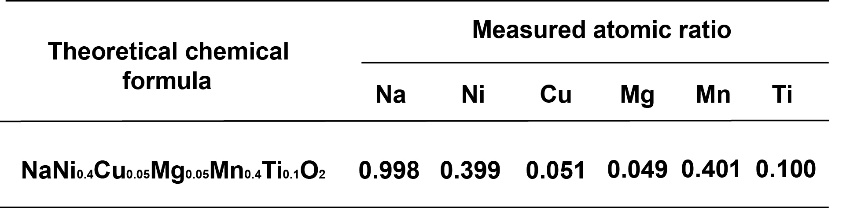


**Table S4.** Specific parameters of electrochemical performance at different rates concerning O3-NaNCMMT electrode in half-cell system.


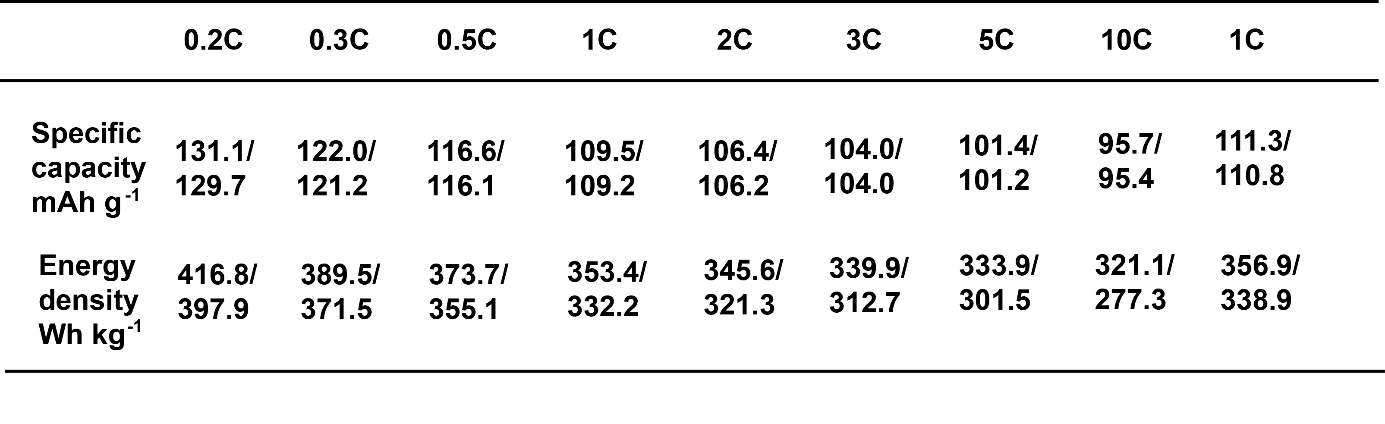


**Table S5.** Summary of the CV results obtained at different scan rates concerning the O3-NaNCMMT cathode material.


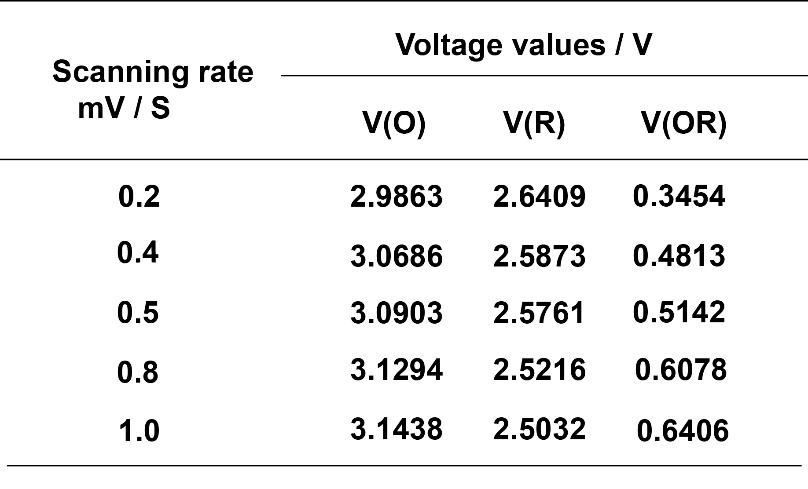


V(O): cathodic peak voltage, V(R): anodic peak voltage, V(OR): the separation between V(O) and V(R).

**Table S6.** Summary of comprehensive performance for various chemical element substituted O3-NaNi_0.5_Mn_0.5_O_2_ cathode materials.


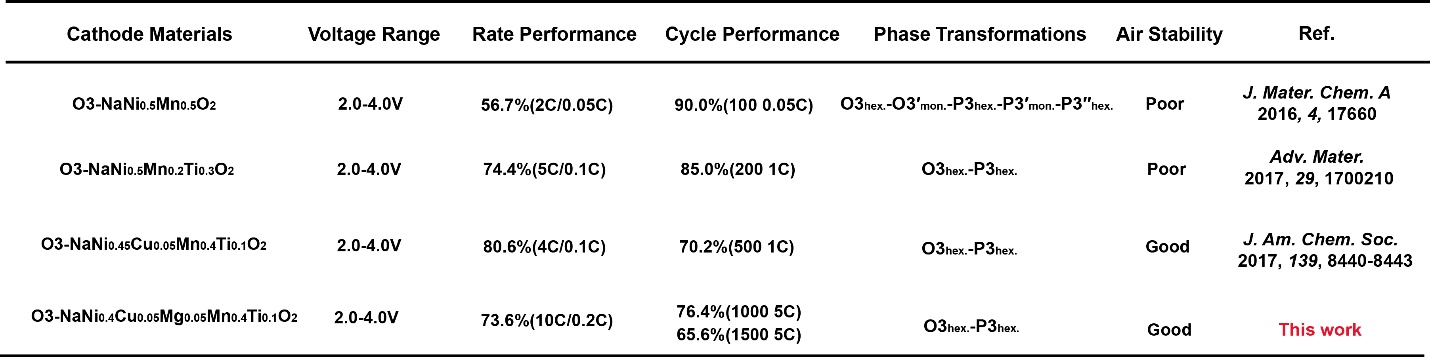
**Table S7.** Summary of the CV results obtained at different scan rates after exposing O3-NaNCMMT cathode material to air for three days.


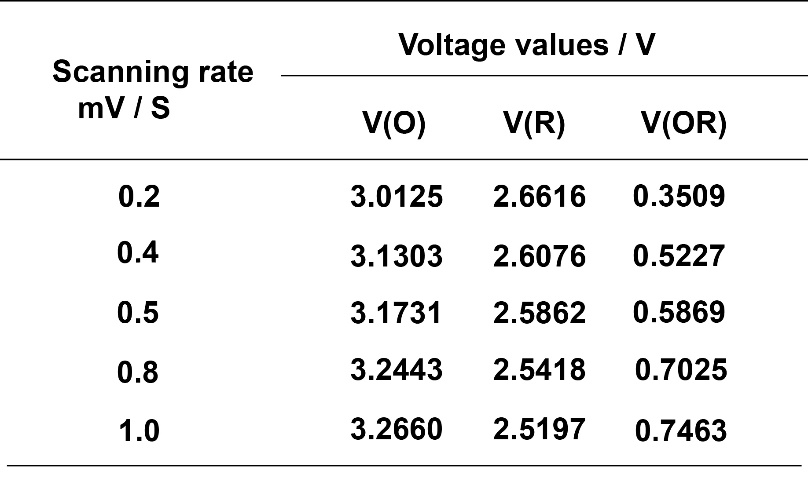


V(O): cathodic peak voltage, V(R): anodic peak voltage, V(OR): the separation between V(O) and V(R).

**Table S8.** Specific parameters of electrochemical performance at different rates concerning O3-NaNCMMT electrode in full-cell system.


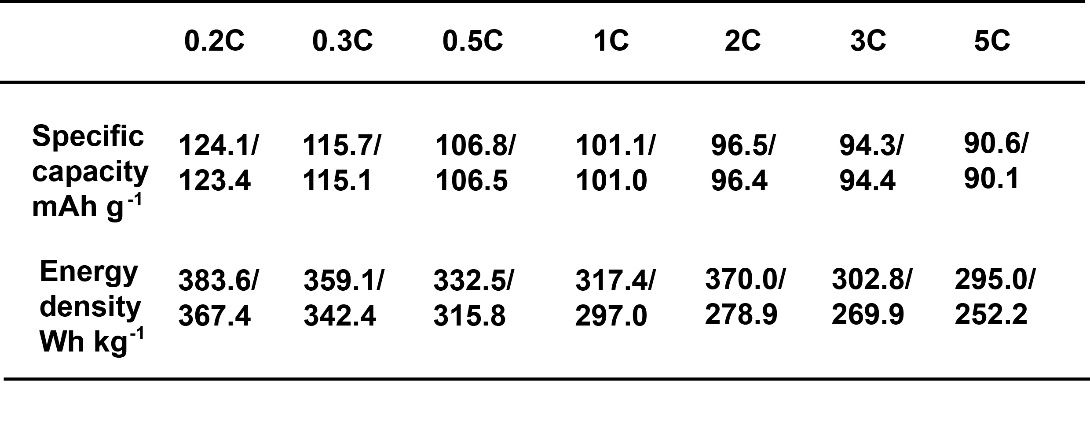

Supplement: Supplementary Materials — Figure S1: crystal structure of O3-NaNCMMT cathode material viewed along the (110) crystallographic direction. Figure S2: optical photograph of precursor concerning O3-NaNCMMT cathode material. Figure S3: (a–c) the colored patterns of HR-TEM image and FFT image as well as line profile of O3-NaNCMMT cathode material viewed along the (010) crystallographic direction. Figure S4: (a–c) the colored patterns of HR-TEM image and FFT image as well as line profile of O3-NaNCMMT cathode material viewed along the (001) crystallographic direction. Figure S5: (a–d) in-situ HEXRD patterns at different temperatures of formation process concerning O3-NaNCMMT cathode material. Figure S6: (a–f): in situ HEXRD patterns at different temperatures of thermal stability concerning O3-NaNCMMT cathode material. Figure S7: (a, b) intensity contour maps (bird's eye view) concerning the evolution of the characteristic diffraction peaks of in-situ HEXRD patterns of formation process and thermal stability concerning O3-NaNCMMT cathode material at different temperatures. Figure S8: (a, b) HAADF and ABF-STEM images and atomic model of O3-NaNCMMT cathode material viewed along the (010) crystallographic direction. Figure S9: (a–d) Typical FFT images and colored patterns of HAADF-STEM images concerning O3-NaNCMMT cathode material viewed along the (010) and (001) crystallographic directions, respectively. Figure S10: EELS spectra of Ti L-edges, O K-edge, Mn L-edges, and Ni L-edges with an increment of 2 nm per spectrum from the surface to the center. Figure S11: electrochemical performance of O3-NaNCMMT electrode in half-cell system. (a) Galvanostatic charge/discharge curves versus specific energy at 0.2C in the voltage range of 2.0-4.0 V. (b) Galvanostatic charge/discharge curves versus specific energy at various rates. (c) Rate performance as the rate comes back to 1C. (d) Linear fitting of the log (i) versus log (v) plots at different oxidation peaks. Figure S12: (a, b) the results of linear fitting [file 1469301.f1.docx]
